# Chromosomal 3q amplicon encodes essential regulators of secretory vesicles that drive secretory addiction in cancer

**DOI:** 10.1172/JCI176355

**Published:** 2024-04-25

**Authors:** Xiaochao Tan, Shike Wang, Guan-Yu Xiao, Chao Wu, Xin Liu, Biyao Zhou, Yu Jiang, Dzifa Y. Duose, Yuanxin Xi, Jing Wang, Kunika Gupta, Apar Pataer, Jack A. Roth, Michael P. Kim, Fengju Chen, Chad J. Creighton, William K. Russell, Jonathan M. Kurie

**Affiliations:** 1 Department of Thoracic/Head and Neck Medical Oncology,; 2 Department of Translational Molecular Pathology, and; 3 Department of Bioinformatics and Computational Biology, The University of Texas MD Anderson Cancer Center, Houston, Texas, USA.; 4 Department of Chemical Sciences, Tata Institute of Fundamental Research, Mumbai, India.; 5 Department of Thoracic and Cardiovascular Surgery and; 6 Department of Surgical Oncology, The University of Texas MD Anderson Cancer Center, Houston, Texas, USA.; 7 Department of Medicine and Dan L Duncan Cancer Center, Baylor College of Medicine, Houston, Texas, USA.; 8 Department of Biochemistry and Molecular Biology, The University of Texas Medical Branch, Galveston, Texas, USA.

**Keywords:** Cell biology, Oncology, Cancer gene therapy, Lung cancer, Protein traffic

## Abstract

Cancer cells exhibit heightened secretory states that drive tumor progression. Here, we identified a chromosome 3q amplicon that serves as a platform for secretory regulation in cancer. The 3q amplicon encodes multiple Golgi-resident proteins, including the scaffold Golgi integral membrane protein 4 (GOLIM4) and the ion channel ATPase secretory pathway Ca^2+^ transporting 1 (ATP2C1). We show that GOLIM4 recruited ATP2C1 and Golgi phosphoprotein 3 (GOLPH3) to coordinate Ca^2+^-dependent cargo loading, Golgi membrane bending, and vesicle scission. GOLIM4 depletion disrupted the protein complex, resulting in a secretory blockade that inhibited the progression of 3q-amplified malignancies. In addition to its role as a scaffold, GOLIM4 maintained intracellular manganese (Mn) homeostasis by binding excess Mn in the Golgi lumen, which initiated the routing of Mn-bound GOLIM4 to lysosomes for degradation. We show that Mn treatment inhibited the progression of multiple types of 3q-amplified malignancies by degrading GOLIM4, resulting in a secretory blockade that interrupted prosurvival autocrine loops and attenuated prometastatic processes in the tumor microenvironment. As it potentially underlies the selective activity of Mn against 3q-amplified malignancies, *ATP2C1* coamplification increased Mn influx into the Golgi lumen, resulting in a more rapid degradation of GOLIM4. These findings show that functional cooperativity between coamplified genes underlies heightened secretion and a targetable secretory addiction in 3q-amplified malignancies.

## Abstract 2

A chromosomal 3q amplicon drives secretion in cancer by encoding essential regulators of secretory vesicles, which can be targeted for therapy using manganese treatment.

## Graphical abstract

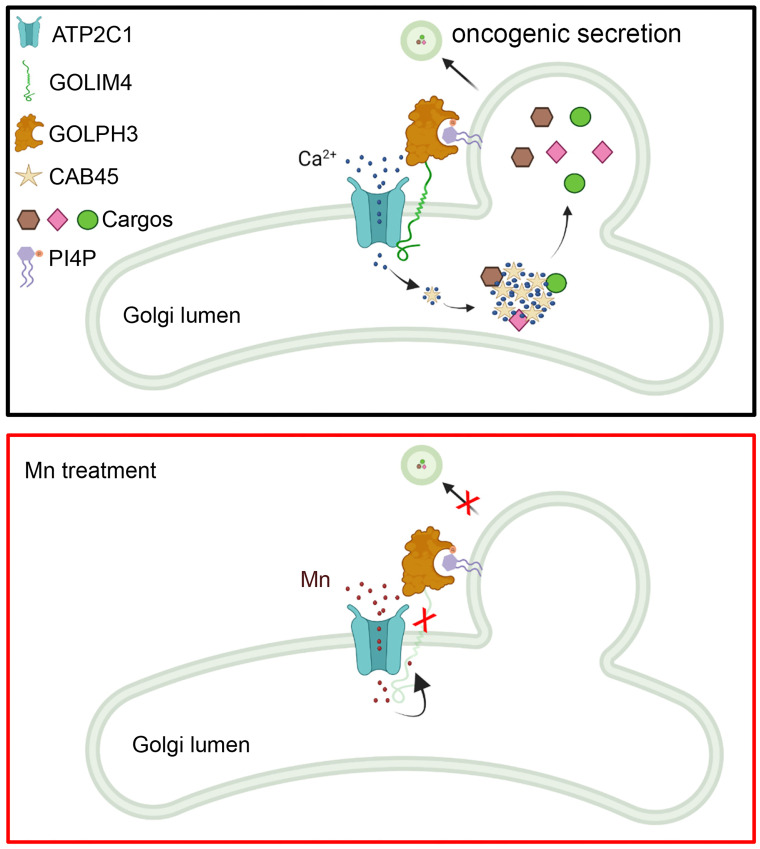

## Introduction

In one working hypothesis, cancer cells are the primary architects of the tumor microenvironment (1). Despite a large body of preclinical evidence supporting their antitumor activities (1), strategies to neutralize matrix metalloproteinases, immune-modulating cytokines, or growth factors in patients have demonstrated limited efficacy in patients with cancer (2). Potentially underlying these outcomes, the cancer secretome is large and functionally redundant (3, 4). Alternative strategies designed to block the entire secretome, rather than the actions of individual secreted proteins, warrant consideration. Such approaches must be based on a thorough understanding of the way in which cancer cells acquire heightened secretory states.

Proteins destined for secretion are transported as vesicular cargos from the endoplasmic reticulum to the plasma membrane via the Golgi apparatus (5). Secretory vesicle biogenesis in the Golgi is a multistep process involving membrane curvature, cargo loading, and vesicle scission; each step is regulated by multiprotein complexes containing RAB family members, ADP ribosylation factors, Golgi phosphoprotein 3 (GOLPH3), and other effectors (6–8). These complexes are anchored to Golgi membranes, in part, by transmembrane Golgi scaffolds that organize client proteins dedicated to a common task (9). Golgi scaffolding proteins upregulated by p53 loss coordinate the actions of secretory drivers in p53-deficient cancer cells (10, 11). Thus, oncogenic mutations drive secretion through Golgi scaffolds that coordinate secretory vesicle biogenesis in the Golgi.

Given the evidence that coamplified genes on chromosomal amplicons function cooperatively to coordinate common biological processes (12), we postulated here that chromosomal amplicons coordinate the multistep process of secretory vesicle biogenesis to establish heightened secretory states. We identified a region of chromosome 3q that is amplified in diverse tumor types and encodes multiple regulators of secretory vesicle biogenesis, including the Golgi scaffold Golgi integral membrane protein 4 (GOLIM4) and its client protein ATPase secretory pathway Ca^2+^ transporting 1 (ATP2C1). Also known as GPP130, GOLIM4 has dual functions: it tethers endosomes to Golgi membranes, and it regulates intracellular manganese (Mn) homeostasis (13). We show that GOLIM4 and ATP2C1 cooperatively drove an addictive secretory process and thereby created a therapeutic vulnerability in 3q-amplified malignancies.

## Results

### GOLIM4 is a protumorigenic effector of the 3q amplicon.

**Figure 1:**
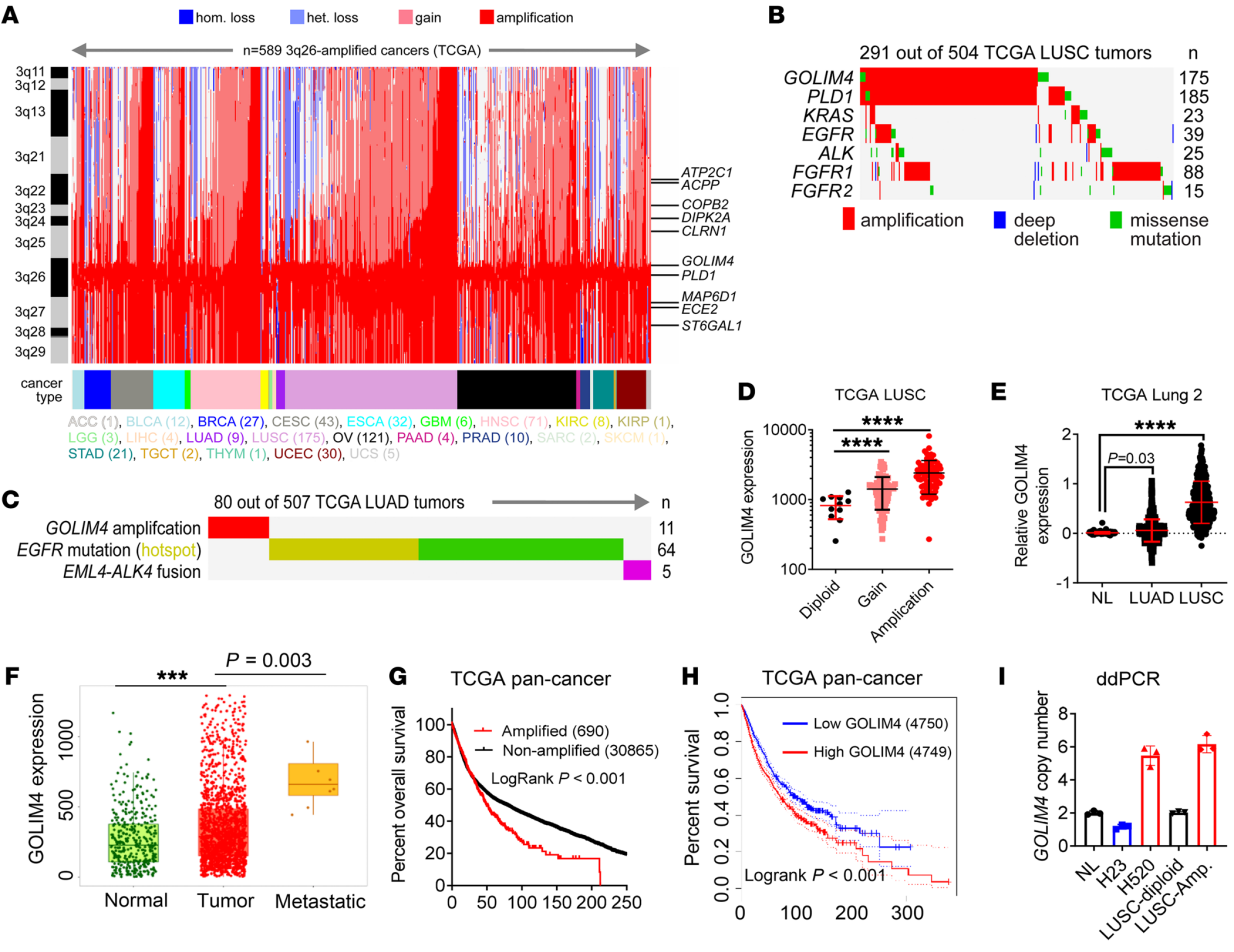
Oncogenomic analysis of the 3q amplicon in human cancers. (**A**) Heatmap of 3q-encoded gene copy numbers (*y* axis) in TCGA pan-cancer cohort (*n* = 589, *x* axis). Genomic regions are color-coded according to the copy number change and tumor type. hom., homozygous; het., heterozygous. (**B** and **C**) Somatic mutations (rows) in TCGA LUSC (**B**) and LUAD (**C**) cohorts (columns). (**D**) Correlation of *GOLIM4* mRNA levels and gene copy numbers in TCGA LUSC samples (data points). Diploid, *n* = 50; gain, *n* = 238; amplification, *n* = 208. (**E**) *GOLIM4* mRNA levels in normal lung tissues (NL) (*n* = 397), LUAD (*n* = 492), and LUSC (*n* = 488). (**F**) *GOLIM4* mRNA levels in NL tissues (*n* = 3,691), primary lung tumors (*n* = 1,865), and distant metastases (*n* = 8) (https://tnmplot.com/). Box plots represent 33% (lower box) and 66% (upper box). *P* values were determined by Dunn’s test. (**G** and **H**) Kaplan-Meier survival analysis of TCGA pan-cancer cohort based on GOLIM4 copy numbers (**G**) and mRNA levels (**H**). (**I**) ddPCR assay of GOLIM4 copy numbers in NL tissues and 3q-amplified (Amp.) and -diploid LUSC tissues. Controls included 3q-amplified (H520) and -diploid (H23) cell lines. Data indicate the mean ± SD from a single experiment incorporating biological replicate samples (*n* = 3, unless otherwise indicated) and are representative of at least 2 independent experiments. ***P < 0.001 and ****P < 0.0001, by 1-way ANOVA (**D**–**F**) or log-rank test (**G** and **H**).

In The Cancer Genome Atlas (TCGA), a region of chromosome 3q is amplified in, among other tumor types, squamous carcinomas of the lung (42%), esophagus (24%), and head and neck (HN) (18%). The minimally amplified region of the 3q amplicon harbors the Golgi scaffold GOLIM4 and the Golgi-resident phospholipase PLD1 (Figure 1A). Outside of this region, the amplicon encodes ATP2C1, a Golgi-resident ion channel (14). Oncogenomic analysis demonstrated negligible convergence of the minimally amplified region with oncogenic driver mutations in lung squamous carcinoma (LUSC) (Figure 1B) or lung adenocarcinoma (LUAD) (Figure 1C). In TCGA lung cancer cohorts, *GOLIM4* mRNA levels were positively correlated with gene copy numbers (Figure 1D), were higher in malignant lung tissues than in normal lung tissues (Figure 1E), and were higher in metastatic deposits than in primary tumors (Figure 1F). *GOLIM4* copy numbers and mRNA levels were correlated with shorter survival durations in TCGA cohorts (Figure 1, G and H). Therefore, we developed a digital droplet PCR (ddPCR) assay to quantify *GOLIM4* copy numbers in genomic DNA samples and showed that GOLIM4 amplification was detectable in LUSC (Figure 1I).

**Figure 2:**
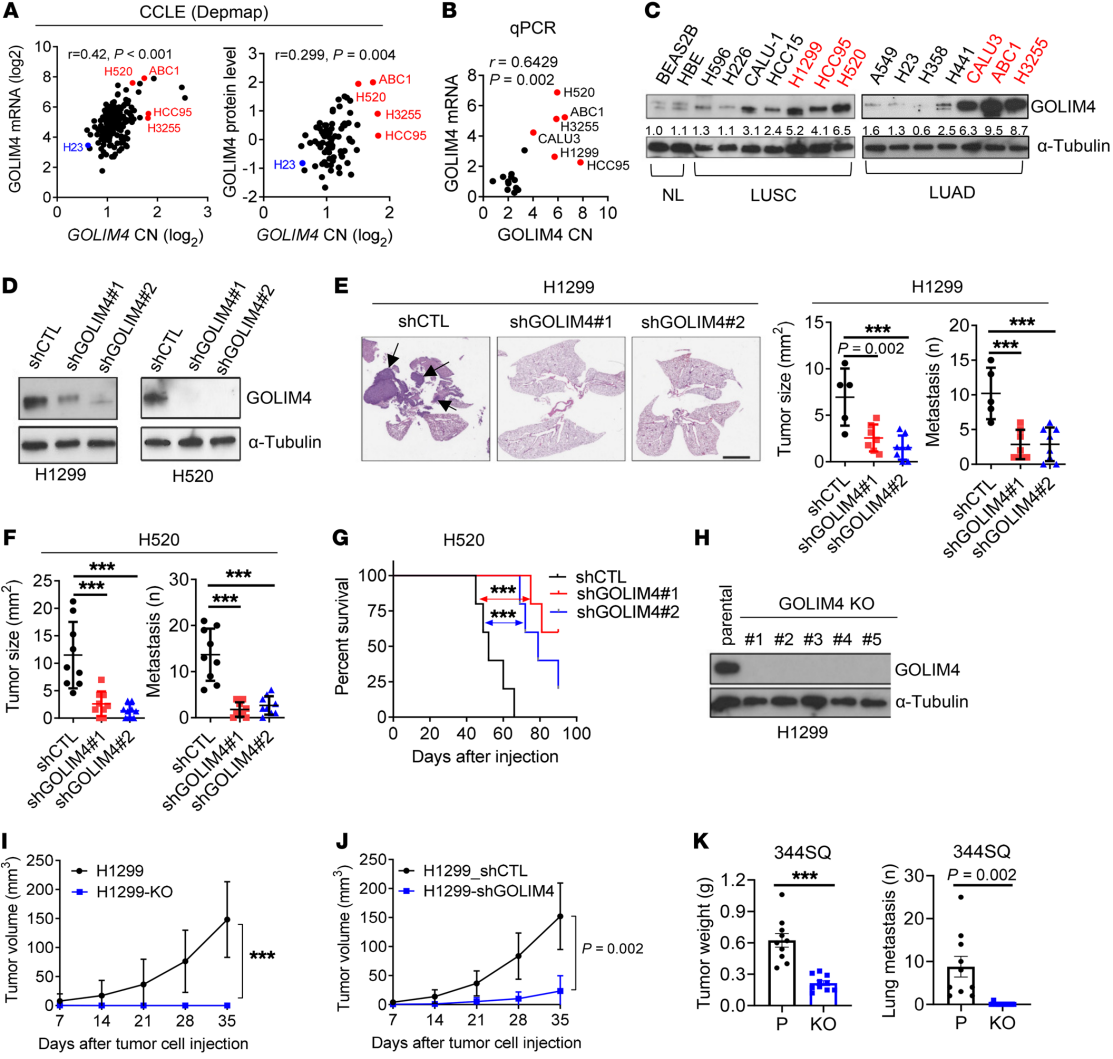
GOLIM4 is a protumorigenic effector of the 3q amplicon. (**A** and **B**) Correlation analyses of 3q-amplified (red) and -diploid (black) lung cancer cell lines. (**A**) *GOLIM4* copy numbers, mRNA levels (left plot), and protein levels (right plot) from the CCLE database. (**B**) qRT-PCR analysis of *GOLIM4* copy numbers (CN) and mRNA levels. (**C**) WB analysis of GOLIM4 protein levels in cell lines. NL, normal lung epithelial cells. (**D**) WB confirmation of target gene depletion in shRNA-transfected H1299 cells. GOLIM4 (shGOLIM4) and control (shCTL) shRNAs were used. (**E** and **F**) Orthotopic lung tumor sizes and distant metastasis numbers generated by shRNA-transfected H1299 cells (**E**) or H520 cells (**F**) and images of H&E-stained lung sections (**E**). Scale bar: 4 mm. Arrows indicate tumors. (**G**) Kaplan-Meier survival analysis of orthotopic H520 tumor–bearing mice. (**H**) WB confirmation of target gene depletion by CRISPR/Cas9-mediated gene editing in H1299 cells. (**I**) Sizes of flank tumors generated by the cells in **H**. Parental (H1299). (**J**) Size of flank tumors generated by the cells in **D**. (**K**) Primary tumor sizes and lung metastasis numbers generated by subcutaneously injected parental and *GOLIM4*-KO 344SQ cells. Data indicate the mean ± SD from a single experiment incorporating biological replicate samples (*n* = 3, unless otherwise indicated) and are representative of at least 2 independent experiments. ***P < 0.001, by 1-way ANOVA (**E** and **F**), log-rank test (**G**), or 2-tailed Student’s *t* test (**I**–**K**).

To test the hypothesis that GOLIM4 is a protumorigenic effector of the 3q amplicon, we first quantified GOLIM4 levels in human lung cancer cell lines classified as 3q amplified or 3q diploid (15) and found that *GOLIM4* mRNA and protein levels were higher in 3q-amplified cells (Figure 2, A–C). We carried out shRNA- or CRISPR/CAS9-mediated *GOLIM4* depletion studies on 3q-amplified human lung cancer cells and found that GOLIM4 deficiency reduced tumor growth and metastatic activity and led to longer survival durations in mice (Figure 2, D–J, and Supplemental Figure 1A; supplemental material available online with this article; https://doi.org/10.1172/JCI176355DS1). GOLIM4-deficient tumors exhibited reduced proliferative activity and higher apoptotic fractions (Supplemental Figure 1, B and C). To examine the role of GOLIM4 in an immunocompetent mouse model, we injected 129/sv mice with a syngeneic LUAD cell line (344SQ) derived from mice that expressed KrasG12D and p53R172H (16). These cells had increased GOLIM4 gene copy numbers (3.5 per cell) (Supplemental Figure 1D). Following CRISPR/Cas-9-mediated *GOLIM4* depletion (Supplemental Figure 1E), 344SQ cells exhibited reduced proliferative and migratory activity in culture and decreased tumor growth and metastatic activity in mice (Figure 2K and Supplemental Figure 1, F and G). In human lung cancer cell lines that were 3q amplified or 3q diploid, GOLIM4 depletion reduced proliferative, migratory, and colony forming activities to a greater extent in 3q-amplified than in 3q-diploid cells (Supplemental Figure 1, H–R). We observed similar findings in breast and HN cancer cell lines (Supplemental Figure 2). Thus, GOLIM4 was a protumorigenic effector of the 3q amplicon.

### GOLIM4 activates protumorigenic secretory processes.

**Figure 3:**
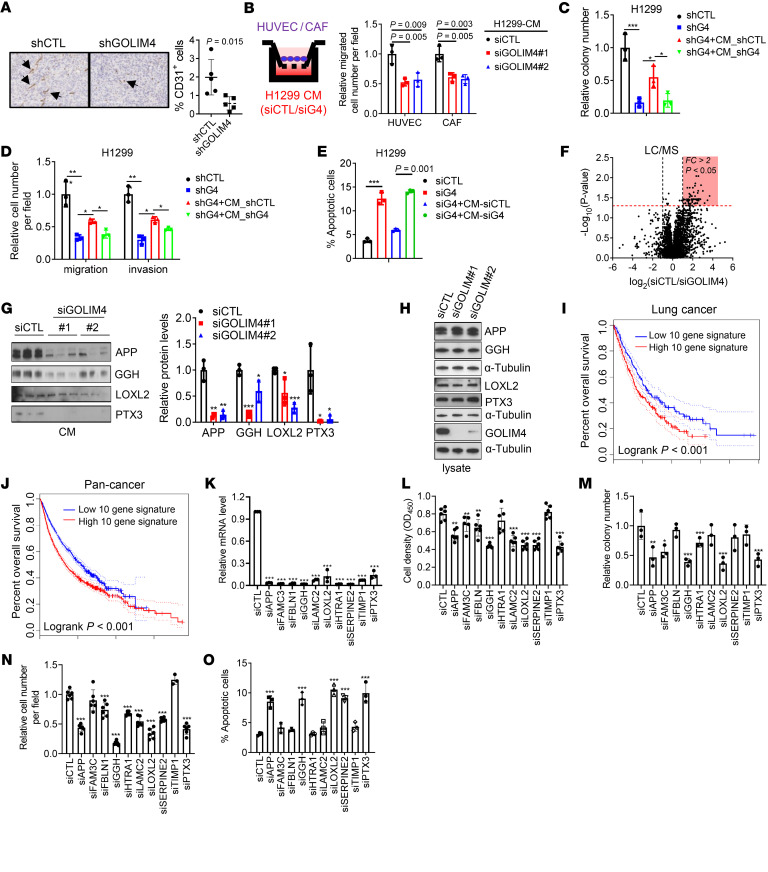
GOLIM4 activates a protumorigenic secretory program. (**A**) Anti-CD31 antibody staining of tumors from Figure 2J. Results are expressed as a percentages of the total numbers of cells analyzed. Original magnification, ×10. Arrows indicate CD31^+^ endothelial cells. (**B**) Boyden chamber assays to quantify the recruitment of HUVECs or CAFs by CM samples from siRNA-transfected H1299 cells. (**C** and **D**) Soft agar colony formation (**C**) and Boyden chamber migration and invasion (**D**) assays on shRNA-transfected H1299 cells treated with CM samples from shRNA-transfected H1299 cells. shGOLIM4 (shG4). Values are expressed relative to the shCTL. (**E**) Annexin V/propidium iodide (PI) flow cytometry of siRNA-transfected H1299 cells to detect apoptosis following treatment with CM samples. (**F**) Volcano plot of proteins identified by LC-MS analysis of CM samples from siRNA-transfected H1299 cells. Results are expressed as a log_2_ ratio (siCTL/siGOLIM4). *y* axis: *P* values; *x* axis: fold change (FC). Proteins downregulated by siGOLIM4 are shown in the shaded areas. (**G** and **H**) WB analysis of proteins identified by LC-MS analysis. CM samples (**G**) and cell lysates (**H**) from siRNA-transfected H1299 cells. Densitometric analysis of bands in **G** expressed relative to siCTL (bar graph). (**I** and **J**) Kaplan-Meier survival analysis of TCGA lung cancer (**I**) and pan-cancer (**J**) cohorts based on a 10-gene signature of GOLIM4-dependent secreted proteins. Tumors were scored as above (high) or below (low) the median signature values for each cohort. *P* < 0.001. (**K**) qRT-PCR confirmation of target gene depletion in siRNA-transfected H1299 cells. (**L**–**O**) Assessment of cells in **K** for relative densities in monolayer culture (**L**), colony formation in soft agar (**M**), invasion in Boyden chambers (**N**), and apoptosis by annexin V/PI flow cytometry (**O**). Data indicate the mean ± SD from a single experiment incorporating biological replicate samples (*n* = 3, unless otherwise indicated) and are representative of at least 2 independent experiments. *P < 0.05, **P < 0.01, and ***P < 0.001, by 2-tailed Student’s *t* test (**A**), 1-way ANOVA (**B**–**E**, **G**, and **K**–**O**), or log-rank test (**I** and **J**).

RNA-Seq studies on GOLIM4-deficient and -replete lung cancer cells showed that GOLIM4-replete cells were relatively enriched in Gene Ontology terms related to secretory processes, including “vesicle cargo loading,” “RAB protein signal transduction,” “monocyte chemotaxis,” and “neuron axon guidance” (Supplemental Figure 3A). Therefore, we postulated that GOLIM4 activates protumorigenic secretory processes and tested this hypothesis by carrying out immunohistochemical studies on GOLIM4-deficient and -replete tumors, which showed that GOLIM4 deficiency reduced endothelial cell numbers in tumor stroma (Figure 3A). In line with these findings, conditioned medium (CM) samples from GOLIM4-replete, 3q-amplified lung cancer cells chemoattracted endothelial cells and fibroblasts and rescued the viability and metastatic properties of GOLIM4-deficient, 3q-amplified cells, whereas CM samples from GOLIM4-deficient cells did not have these effects (Figure 3, B–E, and Supplemental Figure 3, B–E).

To identify GOLIM4-dependent secreted mediators, we performed liquid chromatography–mass spectrometry (LC-MS) analysis on CM samples from 3q-amplified human lung cancer cells that were GOLIM4 deficient or replete and identified a total of 308 proteins, 64 of which were downregulated by GOLIM4 depletion (*P* < 0.05, fold change >1.5; Figure 3F and Supplemental Table 1). Western blot (WB) analysis confirmed that proteins within the downregulated group were secreted in a GOLIM4-dependent manner and showed that GOLIM4 did not regulate the expression levels of these proteins (Figure 3, G and H, and Supplemental Figure 3F). The levels of other secreted proteins identified by LC-MS analysis, such as secreted phosphoprotein 1 and stanniocalcin 2, remained unchanged following GOLIM4 depletion (Supplemental Figure 3G), suggesting that GOLIM4 controlled a specific secretome.

To assess the biological role of GOLIM4-regulated secretion, we selected 10 proteins from the downregulated group that have reported protumorigenic functions (17–26) and were relatively abundant (>10 spectral counts) and found that high expression of the 10-gene signature was correlated with shorter survival durations (Figure 3, I and J). Following siRNA-mediated depletion of several members of the 10-gene signature, including amyloid precursor protein (APP), γ glutamyl hydrolase (GGH), lysyl oxidase-like 2 (LOXL2), or pentraxin 3 (PTX3), 3q-amplified cells exhibited reduced viability, migratory activity, and colony-forming activity (Figure 3, K–O). APP-deficient cells were rescued by APP-replete, but not APP-deficient, CM samples (Supplemental Figure 4, A and B), supporting an on-target effect of the siRNA. Treatment with recombinant APP, GGH, or PTX3 protein partially rescued the viability of GOLIM4-deficient, 3q-amplified cells (Supplemental Figure 4, C–E), suggesting that GOLIM4 activated autocrine signals through these proteins. In line with this conclusion, pharmacologic antagonism or siRNA-mediated depletion of the γ-aminobutyric acid type B receptor 1 (GABBR1), an APP receptor (27), abrogated the ability of recombinant APP to rescue GOLIM4-deficient cells (Supplemental Figure 4, F–J). Conversely, theGABBR1 agonist CGP7930 phenocopied the effects of recombinant APP on GOLIM4-deficient cells (Supplemental Figure 4, G and H). Thus, GOLIM4-dependent secretion activated an APP-dependent autocrine loop that drove tumor progression.

Although GOLIM4 is known to facilitate endosome-to-Golgi trafficking (28), a role in secretory vesicle biogenesis has not been reported, to our knowledge. To address this possibility, we initially assessed GOLIM4 localization and found that it localized in the trans-Golgi network (Supplemental Figure 5A), the site of secretory vesicle biogenesis (5). We quantified cargo loading into secretory vesicles by performing Golgi exit assays on cells transfected with EGFP-tagged APP. Additionally, we quantified the total number of secretory vesicles in cells transfected with fluorescently tagged Rab6A, a secretory vesicle marker. These studies showed that the number of APP^+^ extra-Golgi puncta per cell was reduced in GOLIM4-deficient cells (Supplemental Figure 5B), whereas the total number of Rab6A^+^ vesicles remained unchanged (Supplemental Figure 5C), suggesting that GOLIM4 loss resulted in a cargo-loading defect and an uncoupling of cargo loading from membrane budding and vesicle scission. In temperature-sensitive mutant vesicular stomatitis virus G (VSV-G) assays that quantify cargo loading and anterograde transport (27), we found that plasma membrane–bound VSV-G was reduced in GOLIM4-deficient cells (Supplemental Figure 5D), which is in line with a cargo-loading defect.

**Figure 4:**
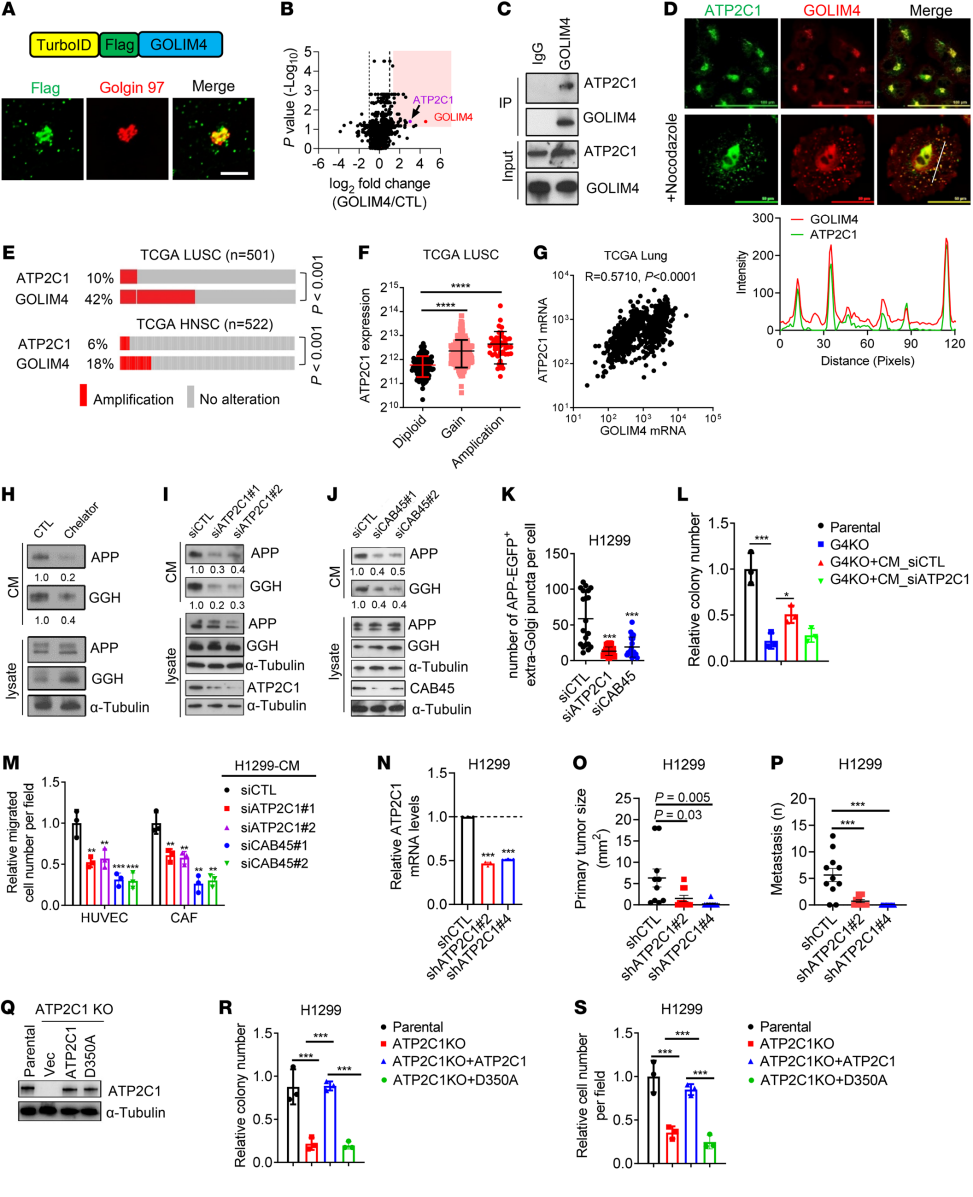
Functional cooperativity between 3q-amplified genes. (**A**) TurboID-GOLIM4 fusion construct (schema, top) localizes in the Golgi (confocal micrographs, bottom). H1299 cells were transfected with TurboID-GOLIM4 and costained with anti-Flag, anti-GM130, and DAPI (blue). Shown are single-channel and merged images. Scale bar: 50 μm. (**B**) Volcano plot of GOLIM4-associated proteins identified by TurboID-based proximity ligation assays. Results for each protein identified (data points) are expressed as a log_2_ ratio (GOLIM4/CTL, *x* axis; *P* value, *y* axis). (**C**) IP/WB confirmation of ATP2C1 as a GOLIM4-associated protein in H1299 cells. (**D**) Confocal micrographs of endogenous ATP2C1 and GOLIM4 in H1299 cells. Cells were treated with nocodazole to disperse the Golgi. Line plot (under images) assesses colocalization of GOLIM4 and ATP2C1. Shown are the signal intensities (*y* axis) and distances from the plasma membrane (*x* axis). (**E**) Gene copy numbers (rows) in tumors (columns). HNSC, HN squamous cell carcinoma. *P* <0.001, significant co-occurrence, by 1-sided Fisher’s exact test. (**F**) Correlations between *ATP2C1* mRNA levels and gene copy numbers in tumors (data points). Diploid, *n* = 109; gain, *n* = 311; amplification, *n* = 48. (**G**) Pearson’s correlation between *GOLIM4* and ATPC1 mRNA levels in tumors (data points). (**H**–**J**) WB analysis of secreted protein levels in CM samples and cell lysates. H1299 cells were treated with a Ca^2+^ chelator (**H**) or transfected with siRNAs against ATP2C1 (**I**) or Cab45 (**J**). (**K**) Number of APP^+^ vesicles in H1299 cells transfected with indicated siRNAs. (**L**) Relative soft agar colony numbers generated by parental and *GOLIM4*-KO (G4KO) H1299 cells following treatment with CM samples from siRNA-transfected H1299 cells. (**M**) Boyden chamber assays to quantify HUVEC and CAF recruitment by CM samples from siRNA-transfected H1299 cells. (**N**) qRT-PCR confirmation of target gene depletion by ATP2C1 shRNAs in H1299 cells. (**O** and **P**) Orthotopic tumor size (**O**) and distant metastases (**P**) per mouse (data points) generated by H1299 transfectants in **N**. (**Q**) WB confirmation of ATP2C1 reconstitution in *ATP2C1*-KO cells by WT or D350A-mutant ATP2C1. Vec, empty vector. (**R** and **S**) Soft agar colony formation assay (**R**) and Boyden chamber migration assay (**S**) on cells generated in **Q**. Data indicate the mean ± SD from a single experiment incorporating biological replicate samples (*n* = 3, unless otherwise indicated) and are representative of at least 2 independent experiments. *P < 0.05, **P < 0.01, ***P < 0.001, and ****P < 0.0001, by 1-way ANOVA (**F**, **K**–**P**, **R**, and **S**).

To elucidate how GOLIM4 regulates cargo loading, we carried out proximity ligation assays utilizing a TurboID-GOLIM4 fusion protein as bait (Figure 4A). LC-MS analysis of the resultant biotinylated proteins identified a total of 47 candidate interactors, including ATP2C1 (Figure 4B and Supplemental Table 2), which was confirmed to be a GOLIM4-associated protein by immunoprecipitation/WB (IP/WB) analysis and coimmunofluorescence studies (Figure 4, C and D). ATP2C1 is a Golgi-resident Ca^2+^/Mn^2+^ channel that interacts with cofilin1 and F-actin at the cytosolic interface to promote Ca^2+^ influx into the Golgi lumen, which causes Ca^2+^-binding protein 45 kDa (CAB45) to oligomerize, bind secretory cargoes, and initiate sorting of Cab45-cargo complexes into secretory vesicles (14, 29). Located on the 3q amplicon (Figure 1A), *ATP2C1* is coamplified with GOLIM4 (Figure 4E), and *ATP2C1* mRNA levels are correlated with *ATP2C1* gene copy numbers and *GOLIM4* mRNA levels in TCGA lung cancer cohorts (Figure 4, F and G).

To assess the role of the ATP2C1/CAB45 axis in GOLIM4-dependent secretion, we first conducted co-IP assays and found that APP and GGH were components of a CAB45-containing protein complex (Supplemental Figure 6A). Inhibition of Ca^2+^-dependent secretion by treatment with Ca^2+^ chelators decreased APP and GGH secretion (Figure 4H and Supplemental Figure 6B). Therefore, we used siRNA-mediated ATP2C1 depletion as a tool to block Ca^2+^ influx into the Golgi lumen, which was confirmed using a Golgi-localized Ca^2+^ reporter as a readout of intraluminal Ca^2+^ concentrations (Supplemental Figure 6C). We found that depletion of ATP2C1 or CAB45 attenuated APP and GGH secretion (Figure 4, I and J) and reduced the number of APP^+^ extra-Golgi puncta per cell (Figure 4K). Moreover, ATP2C1 depletion phenocopied the effect of GOLIM4 depletion in 3q-amplified lung cancer cells (Figure 4, L–P, and Supplemental Figure 6, D–K). An ATP2C1 mutant that lacks Ca^2+^ transporter activity failed to rescue ATP2C1-deficient cells (Figure 4, Q–S). These findings support the conclusion that ATP2C1 is a GOLIM4 client that activates Ca^2+^-dependent cargo sorting to drive secretion in 3q-amplified lung cancer.

**Figure 5:**
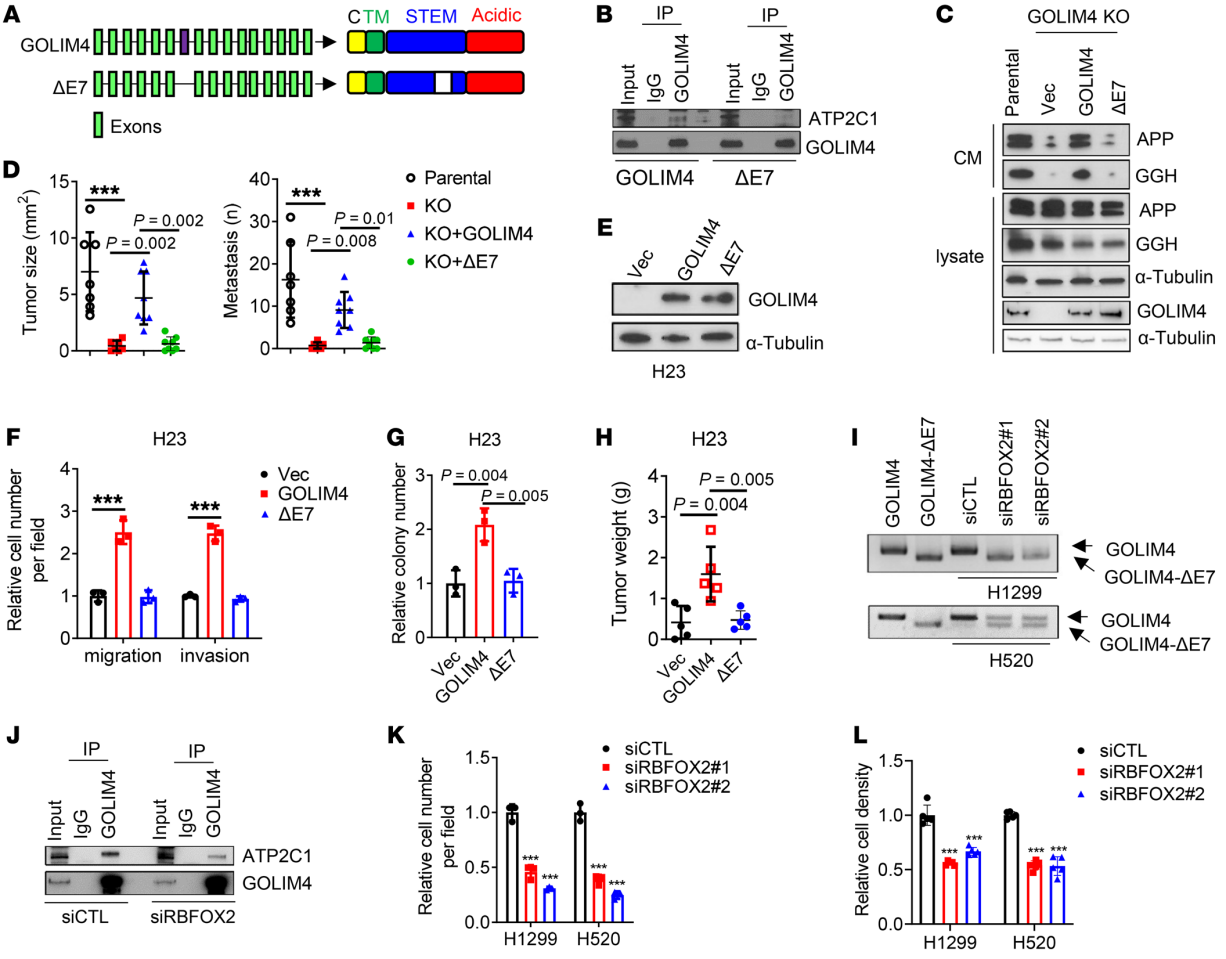
An alternatively spliced exon in *GOLIM4* is required for ATP2C1-binding and secretory activities. (**A**) Full-length and spliced (ΔE7) *GOLIM4* isoforms. Exon 7 is located in the intraluminal STEM domain. TM, transmembrane domain; C, cytoplasmic domain. (**B**) IP/WB analysis of GOLIM4-KO H1299 cells reconstituted with full-length or ΔE7 GOLIM4. ATP2C1-binding activity was detected only in full-length GOLIM4-transfected cells. (**C**) WB analysis of secreted proteins in CM samples and cell lysates from parental and *GOLIM4*-KO H1299 cells reconstituted with full-length or ΔE7 GOLIM4. α-Tubulin was used as a loading control. (**D**) Quantification of orthotopic tumor size (left) and distant metastases (right) per mouse (data points) generated by H1299 cells in **C**. (**E**) WB analysis of GOLIM4 levels in H23 cells transfected with full-length GOLIM4 or GOLIM4-ΔE7. (**F**–**H**) Boyden chamber migration and invasion assays (**F**), soft agar colony assays (**G**), and flank tumor growth assays (**H**) on cells in **E**. (**I**) qRT-PCR analysis of *GOLIM4* isoforms in siRNA-transfected H1299 and H520 cells. Full-length GOLIM4 and GOLIM4-ΔE7 were included as controls. (**J**) IP/WB analysis of H1299 cells demonstrates that FOXF2 depletion attenuated the ATP2C1-binding activity of GOLIM4. (**K** and **L**) Boyden chamber migration assays (**K**) and relative cell density assays (**L**) on siRNA-transfected H1299 and H520 cells. Data indicate the mean ± SD from a single experiment incorporating biological replicate samples (*n* = 3, unless otherwise indicated) and are representative of at least 2 independent experiments. ***P < 0.001, by 1-way ANOVA (**D**, **F**–**H**, **K**, and **L**).

*GOLIM4* exon 7 encodes a portion of the intraluminal domain and is alternatively spliced (30). To determine whether this region interacts with ATP2C1, we performed serial deletion studies on GOLIM4 and found that ATP2C1-binding activity mapped to the intraluminal domain (Supplemental Figure 7A). A long form of *GOLIM4* that includes the alternatively spliced exon is detectable in 3q-amplified cancer cells and is reduced following depletion of RBFOX2, which drives inclusion of exon 7 (30) (Figure 5A). In reconstitution studies carried out on GOLIM4-deficient cells, a short form of GOLIM4 that lacked the alternatively spliced exon (GOLIM4-ΔE7) did not bind to ATP2C1 (Figure 5B and Supplemental Figure 7B) or rescue secretory, tumorigenic, or metastatic activities of GOLIM4-deficient lung cancer cells (Figure 5, C–H). In line with these findings, RBFOX2 depletion attenuated GOLIM4-ATP2C1 interactions (Figure 5, I and J, and Supplemental Figure 7C) and reduced the migratory and proliferative activities of 3q-amplified lung cancer cells (Figure 5, K and L). Thus, the intraluminal domain of GOLIM4 interacted with ATP2C1 to initiate secretion.

**Figure 6:**
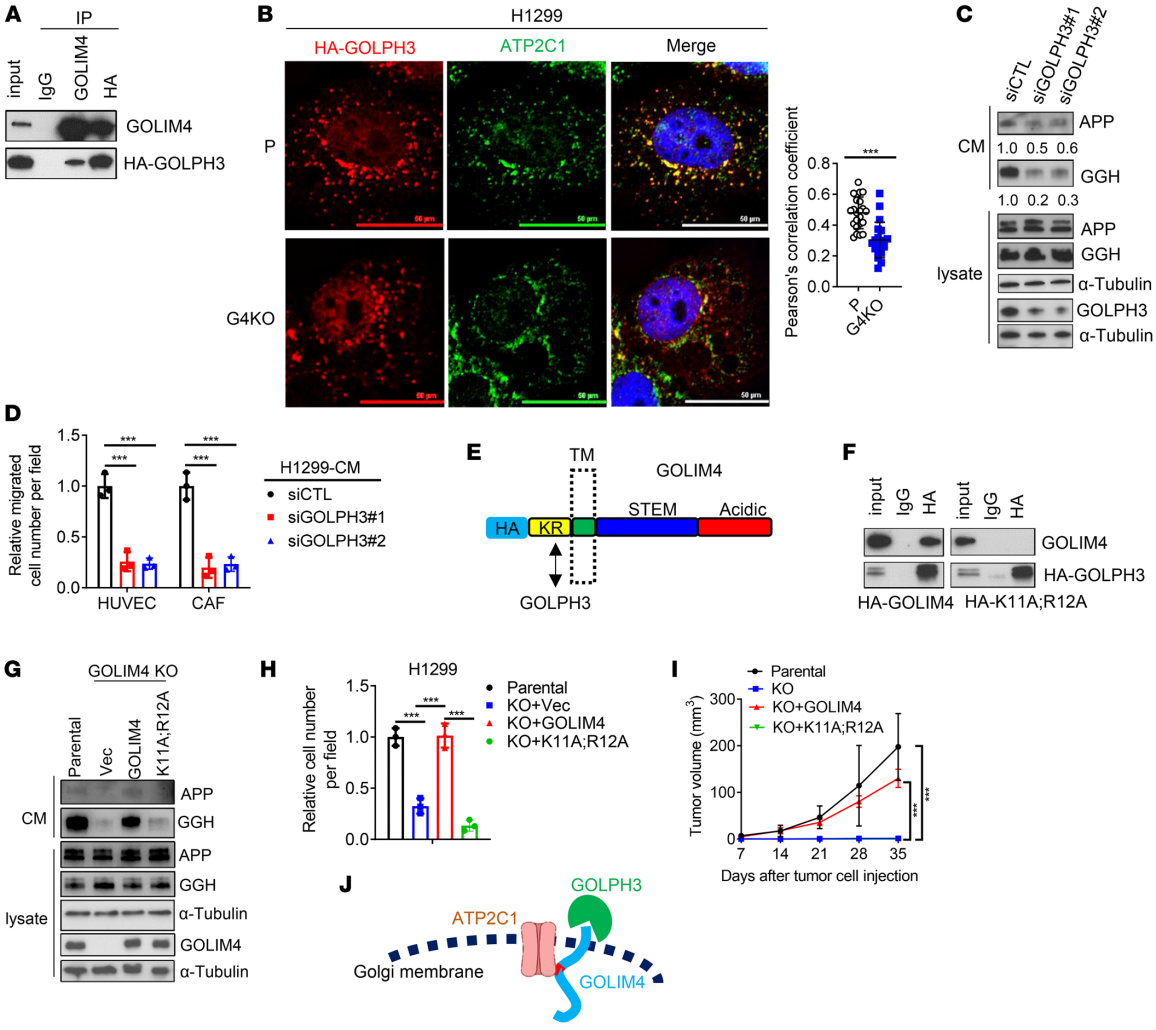
GOLPH3 is a GOLIM4 client protein that promotes secretion. (**A**) IP/WB analysis of H1299 cells detected HA-GOLPH3 in a GOLIM4-containing protein complex. (**B**) Confocal micrographs of H1299 cells transfected with HA-GOLPH3. Cells were treated with nocodazole to disperse the Golgi and costained with anti-HA and anti-ATP2C1. Scatter plot shows the percentages of HA-GOLPH3 that colocalized with ATP2C1 in each cell (data points). Scale bars: 50 μm. (**C**) WB analysis of secreted protein levels in CM samples and cell lysates following siRNA-mediated GOLPH3 depletion. Relative densitometric values under the gel lanes. α-Tubulin was used as a loading control. (**D**) Boyden chamber assays to quantify HUVEC and CAF recruitment by CM samples from siRNA-transfected H1299 cells. (**E**) GOLIM4 domain structure. KR residues are required to bind to GOLPH3. HA tag, luminal stem (STEM), and acidic domains are shown in the schema. (**F**) IP/WB analysis of *GOLIM4*-KO H1299 cells reconstituted with HA-tagged WT (GOLIM4) or mutant (K11A;R12A) GOLIM4. (**G**) WB analysis of secreted proteins in CM samples and lysates from *GOLIM4*-KO H1299 cells reconstituted with WT or mutant GOLIM4. (**H** and **I**) Boyden chamber migration assays (**H**) and flank tumor growth assays (**I**) on cells generated in **G**. (**J**) ATP2C1 and GOLPH3 are clients of the GOLIM4 scaffold. Data indicate the mean ± SD from a single experiment incorporating biological replicate samples (*n* = 3, unless otherwise indicated) and are representative of at least 2 independent experiments. ***P < 0.001, by 2-tailed Student’s *t* test (**B**) or 1-way ANOVA (**D**, **H**, and **I**).

Secretory vesicle biogenesis requires Golgi membrane bending, budding, and scission (5). GOLPH3, which bridges Golgi membranes to actomyosin fibers that initiate membrane bending (6, 7), was recently identified as a client protein of GOLIM4 (31). We found that GOLPH3 was a component of a GOLIM4-containing protein complex (Figure 6A) and that GOLIM4 was required to maintain the integrity of that complex (Figure 6B). GOLPH3 depletion reduced secretion and the chemotactic activity of CM samples derived from 3q-amplified cells (Figure 6, C and D). Reconstitution of GOLIM4-deficient cells with a mutant GOLIM4 lacking cytosolic domain residues (KR) required to bind GOLPH3 (31) did not rescue secretory, colony-forming, or tumorigenic activities (Figure6, E–J). Thus, GOLIM4 exerted broad control of secretory vesicle biogenesis through clients that govern cargo loading and Golgi membrane bending.

### Mn degrades GOLIM4 to target a secretory dependency in 3q-amplified malignancies.

In addition to functioning as a scaffold, GOLIM4 maintains intracellular Mn homeostasis by binding Mn in the Golgi lumen to generate Mn-bound GOLIM4 oligomers that are transported to lysosomes and degraded (32). To determine whether GOLIM4 functions as a Mn-binding protein in 3q-amplified cancer cells, we passed cell lysates over a Mn-binding resin and conducted LC-MS analysis on the eluted proteins, which identified a total of 10 proteins, including GOLIM4, the only Golgi-resident protein with detectable Mn-binding activity (Supplemental Figure 8, A and B). Mn treatment caused GOLIM4 to translocate to lysosomes and undergo degradation, which was blocked by lysosomal, but not proteasomal, inhibitors (Supplemental Figure 8, C and D).

To elucidate how GOLIM4 lysosomal translocation occurs, we carried out proximity ligation assays on Mn-treated cells utilizing the TurboID-GOLIM4 fusion protein as bait (Supplemental Figure 9A). By LC-MS analysis of the resultant biotinylated proteins, we identified a total of 156 candidate interactors, including, among other proteins, the BAG6-UBL4A-GET4 protein complex, which regulates membrane protein targeting and protein quality control (33), and GGA1, a member of a Golgi-localized, γ adaptin ear–containing, ARF-binding protein family that mediates transmembrane receptor sorting on the trans-Golgi network (34) (Supplemental Figure 9A and Supplemental Table 3). After confirming by co-IP studies that Mn causes the BAG6 complex and GGA1 to associate with GOLIM4 (Supplemental Figure 9B), we performed siRNA-mediated depletion studies of these GOLIM4-associated proteins and found that Mn-induced GOLIM4 degradation was mitigated only by GGA1 depletion (Supplemental Figure 9, C and D). In line with these findings, Mn caused GOLIM4 to translocate into GGA1-containing vesicles (Supplemental Figure 9E), and GGA1 depletion prevented GOLIM4 from leaving the Golgi and accumulating in lysosomes (Supplemental Figure 9, F and G). Thus, Mn-bound GOLIM4 oligomers recruited GGA1 to initiate GOLIM4 translocation to lysosomes.

**Figure 7:**
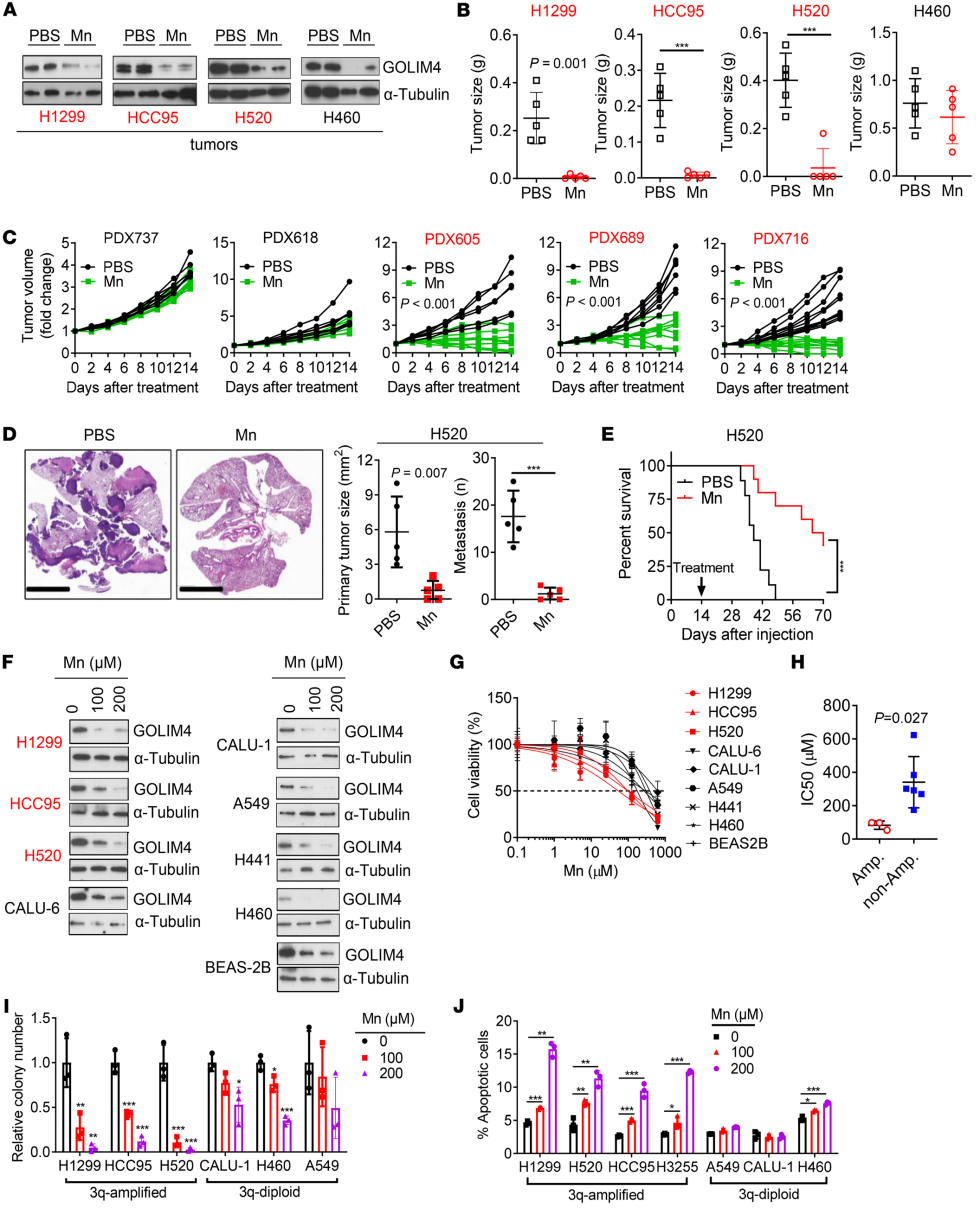
Mn treatment downregulates GOLIM4 levels and exerts selective antitumor activity in 3q-amplified lung cancer. (**A**–**C**) WB analysis of intratumoral GOLIM4 levels (**A**) and flank tumor weights generated by 3q-amplified (red) or -diploid (black) lung cancer cell lines (**B**) or LUSC PDX models (**C**). Prior to sacrifice, mice were treated for 2 weeks with Mn or PBS. (**D**) H&E staining of lung sections. Scale bars: 5 mm. Orthotopic tumor size (left plot) and metastasis numbers (right plot) generated by H520 cells. (**E**) Kaplan-Meier survival analysis of orthotopic H520 orthotopic tumor–bearing mice treated with Mn or PBS. (**F**) WB analysis of GOLIM4 levels in lung cancer cell lines treated with Mn. Immortalized human bronchial epithelial cells (BEAS-2B) were included as a control. (**G**) Relative densities of lung cancer cell lines treated for 4 days with different doses of Mn. (**H**) IC_50_ values from **G**. (**I** and **J**) Soft agar colony formation assays (**I**) and annexin V/PI flow cytometry–based apoptosis assays (**J**) on lung cancer cells treated with Mn. Data indicate the mean ± SD from a single experiment incorporating biological replicate samples (*n* = 3, unless otherwise indicated) and are representative of at least 2 independent experiments. ***P < 0.001, by 2-tailed Student’s *t* test (**B**–**D**, and **H**), 2-way ANOVA (**I** and **J**), or log-rank test (**E**).

On the basis of these findings, we reasoned that Mn might be utilized as a targeted therapy for 3q-amplified malignancies and addressed this possibility by treating tumor-bearing mice with a Mn dose shown to be well tolerated (35). Under these conditions, Mn treatment induced no weight loss or histologic evidence of damage to major organs (Supplemental Figure 10, A and B). Mn decreased intratumoral GOLIM4 levels irrespective of 3q amplification status but exerted greater proapoptotic and antitumor activity in 3q-amplified than 3q-diploid models, including tumors generated by lung cancer cell lines or lung or pancreatic cancer patient–derived xenografts (PDXs) (Figure 7, A–E, and Supplemental Figure 10, C–E). In line with these findings, Mn treatment led to greater activity against 3q-amplified than 3q-diploid lung, breast, and HN cancer cell lines in proliferation, apoptosis, and colony formation assays (Figure 7, F–J, and Supplemental Figure 10, F and G). Following Mn treatment, tumors generated by murine 344SQ cells in syngeneic, immunocompetent mice were smaller and less metastatic (Supplemental Figure 10H), had higher apoptotic fractions (Supplemental Figure 10I), and recruited fewer endothelial cells and fibroblasts (Supplemental Figure 10, J and K). These findings are in line with evidence that GOLIM4 depletion in tumor cells inhibited endothelial cell and fibroblast chemotaxis (Figure 3B).

To compare the efficacy of Mn with that of a proven targeted therapy, we used H3255 cells, which harbor a 3q amplicon and an activating EGFR mutation (L858R). Following H3255 cell injection, mice were treated daily with Mn or the EGFR antagonist osimertinib, and tumors were measured during and after discontinuation of treatment, which showed that tumors regressed to a similar degree following treatment with either drug (Supplemental Figure 10L). However, tumors recurred following discontinuation of Mn but not osimertinib (Supplemental Figure 10L), suggesting that the Mn-induced antitumor effect was reversible. Reversible growth arrest can result from mitochondrial stress (36), which is reported to occur with Mn treatment (37). However, we found that mitochondrial membrane integrity remained intact in Mn-treated cells (Supplemental Figure 11, A–C), arguing against mitochondrial stress as a contributor to the Mn-induced growth arrest. Instead, findings from VSV-G trafficking assays (Supplemental Figure 11D), CM transfer studies (Supplemental Figure 11, E and F), and CM proteomics analysis (Supplemental Figure 11, G–I) showed that Mn treatment caused a secretory blockade similar to that induced by GOLIM4 depletion.

Given that multiple Mn-binding proteins were identified in 3q-amplified lung cancer cells (Supplemental Figure 8B), we asked whether Mn exerts antitumor activity through GOLIM4-independent mechanisms. Arguing against this possibility, GOLIM4 depletion reduced the proapoptotic and antitumor effects of Mn treatment on 3q-amplified lung cancer cells (Supplemental Figure 11, J–L). Furthermore, in GOLIM4-deficient cells reconstituted with a GOLIM4 mutant that does not bind to Mn (Supplemental Figure 11M), Mn did not degrade GOLIM4, inhibit secretion, decrease tumor cell viability or migration, or suppress tumor growth or metastasis (Supplemental Figure 11, N–S). Thus, the antitumor effects of Mn were directly tied to GOLIM4.

**Figure 8:**
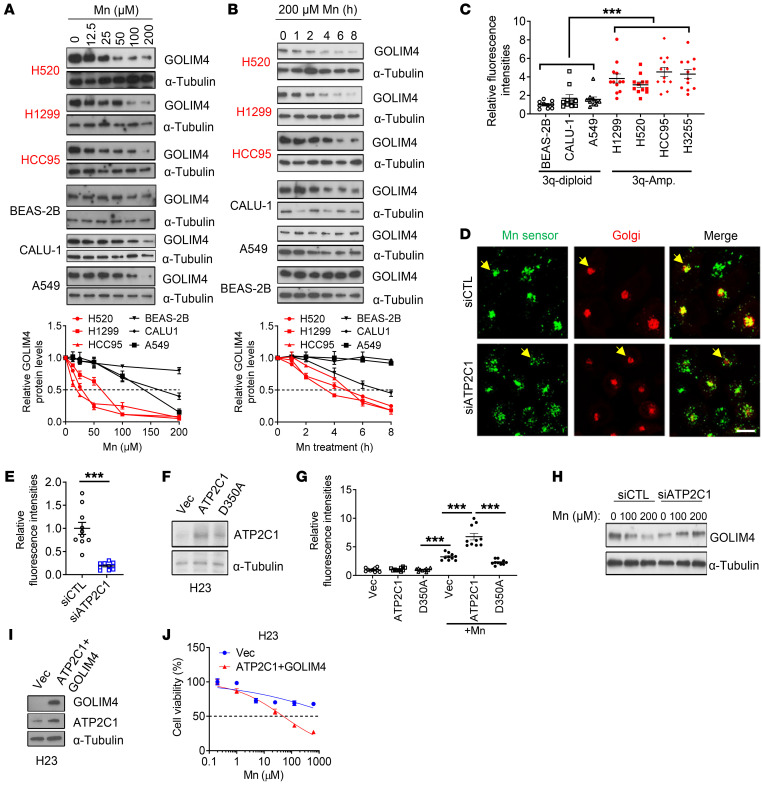
ATP2C1 sensitizes 3q-amplified lung cancer cells to Mn. (**A** and **B**) WB analysis of GOLIM4 levels in lung cancer cells treated with Mn in a dose-dependent (**A**) or time-dependent (**B**) manner. Densitometric quantification of GOLIM4 (line graphs). Values normalized to PBS-treated cells. (**C**) Intra-Golgi Mn sensor M1 activity in lung cancer cells (data points) treated with Mn. (**D**) Single-channel and merged confocal micrographs of Mn sensor M1 (green) in siRNA-transfected H1299 cells treated with Mn. CellLight Golgi-RFP staining is shown in red. Scale bar: 100 μm. (**E**) Relative Mn sensor M1 intensity per cell (data points) in **D**. (**F**) WB analysis of H23 cells stably transfected with WT (ATP2C1) or D350A-mutant ATP2C1. (**G**) Relative Mn sensor M1 intensity per cell (data points) after Mn treatment of cells generated in **F**. (**H**) WB analysis of GOLIM4 levels in siRNA-transfected H1299 cells after Mn treatment. (**I**) WB analysis of H23 cells cotransfected with GOLIM4 and ATP2C1. (**J**) Relative densities of H23 cells in **I** treated with Mn. Data indicate the mean ± SD from a single experiment incorporating biological replicate samples (*n* = 3, unless otherwise indicated) and are representative of at least 2 independent experiments. ***P < 0.001, by 2-tailed Student’s *t* test (**C**, **E**, and **J**) or 1-way ANOVA (**G**).

While Mn induced GOLIM4 degradation irrespective of 3q amplification status (Figure 7F), GOLIM4 degradation occurred more rapidly in 3q-amplified than 3q-diploid lung cancer cells (Figure 8, A and B). ATP2C1 transports both Ca^2+^ and Mn, which led us to speculate that *ATP2C1* amplification increases intra-Golgi Mn levels to accelerate GOLIM4 degradation. To address this possibility, we used a reporter assay that quantifies intra-Golgi Mn levels (38, 39) and showed that reporter activity was higher in 3q-amplified than 3q-diploid cells and was reduced by ATP2C1 depletion in 3q-amplified cells (Figure 8, C–E, and Supplemental Figure 12). Ectopic expression of WT ATP2C1 increased Mn reporter activity in a 3q-diploid cell line, whereas a mutant ATP2C1 that lacked transporter activity did not have this effect (Figure 8, F and G). ATP2C1 depletion abrogated Mn-induced GOLIM4 degradation in 3q-amplified cells (Figure 8H), and ectopic coexpression of GOLIM4 and ATP2C1 sensitized a 3q-diploid lung cancer cell line (H23) to Mn treatment (Figure 8, I and J). These findings support the conclusion that *ATP2C1* coamplification increased intra-Golgi Mn levels to hasten GOLIM4 degradation and enhanced the antitumor effects of Mn treatment in 3q-amplified cancer cells.

## Discussion

The Golgi apparatus is a complex network of membrane-bound compartments that process and sort proteins and lipids (5). Golgi scaffolds play a crucial role in maintaining the structure and function of the Golgi apparatus within cells (40, 41). For example, scaffolds organize cisternae into stacks that are essential for the proper processing and sorting of proteins and lipids (9, 42). Scaffolds also regulate the size and shape of the Golgi apparatus and are involved in vesicle tethering, fusion, and transport (40, 41, 43). More recently, scaffolds have emerged as important regulators of cancer growth and metastasis. We previously showed that the scaffolds progestin and adiponectin Q receptor 11 (PAQR11) and Golgi reassembly and stacking protein 55kD (GRASP55) promote lung cancer growth and metastasis (10, 11). PAQR11 maintains a compact and polarized Golgi structure in lung cancer cells that have undergone epithelial-mesenchymal transition and recruits ARF1 to the Golgi membrane to facilitate vesicle formation and secretion (41). GRASP55, which plays an essential role in organizing Golgi cisternae into a stacked structure, forms a multiprotein complex that regulates the secretion of protumorigenic proteins (10). In this study, we investigated the role of GOLIM4 in 3q-amplified malignancies and found that GOLIM4 recruited client proteins that coordinated secretory vesicle biogenesis and activated a heightened secretory state.

Although mechanisms of secretory cargo sorting are not yet fully understood, sorting signals on cargo proteins and specific lipids within Golgi membranes play crucial roles (5). Sorting signals are recognized by specific receptors or adapters on Golgi membranes, which recruit cargo into vesicles targeted to specific compartments (5, 44). Lipids, such as phosphoinositides and sphingolipids, also act as recognition signals for cargo sorting (45, 46). For the ATP2C1/CAB45-mediated, Ca^2+^-dependent cargo-sorting pathway, Ca^2+^ influx into the Golgi lumen leads to the aggregation of Ca^2+^-binding protein CAB45, which binds to soluble proteins and directs their sorting into vesicles (14, 29). Our study revealed that GOLIM4 regulated secretion through this pathway. However, elucidating the ways in which the CAB45 protein complex selects specific cargos will require further investigation.

We show that GOLIM4 activated a protumorigenic secretory process and that Mn treatment depleted GOLIM4 and inhibited the loading and secretion of GOLIM4-dependent cargos. Underlying the secretory blockade induced by Mn treatment, several findings suggest that GOLIM4 depletion disrupts scaffolding functions that coordinate key drivers of secretory vesicle biogenesis. First, ATP2C1 and GOLPH3 are GOLIM4 clients that colocalized in the Golgi, owing to scaffolding functions of GOLIM4. Second, ATP2C1 was coamplified with GOLIM4, which resulted in high intra-Golgi Ca^2+^ levels that activated a Ca^2+^-dependent cargo-sorting process through ATP2C1 and its effector CAB45. Third, GOLPH3, ATP2C1, and CAB45 were required for GOLIM4-dependent cargo loading and a protumorigenic secretory process activated by GOLIM4. Fourth, a GOLIM4 splice variant that cannot bind ATP2C1 did not enhance secretion or promote tumor growth, linking ATP2C1-binding activity to a protumorigenic secretory process activated by GOLIM4. Fifth, a GOLIM4 mutant that cannot bind Mn did not rescue the antitumor effects of Mn in GOLIM4-deficient tumor cells, linking GOLIM4 to Mn-induced blockade of a protumorigenic secretory process.

The underlying basis for the selective activity of Mn against 3q-amplified malignancies remains unclear, but our data point to *ATP2C1* coamplification as a potential contributor. High ATP2C1 levels increased intra-Golgi Mn concentrations, which hastened GOLIM4 degradation and resulted in secretory blockade. Multiple GOLIM4-dependent secreted proteins maintained the viability, motility, invasion, and colony-forming activities of cancer cells, and Mn treatment inhibited the secretion of key effectors. Reconstituting those effectors through CM transfer reversed Mn-induced cell death and loss of colony-forming activity, linking the 3q amplicon to an addictive secretory process.

Metal-based compounds, including platinum-based drugs and gold-based compounds, have shown promise in cancer therapy (47). These compounds can selectively interact with cancer cells or specific molecular targets to cause DNA damage, generate ROS, and inhibit crucial enzymes (47, 48). Compared with conventional chemotherapy, metal-based targeted therapy offers potential advantages in terms of specificity and reduced toxicity (48). However, challenges remain in optimizing selectivity, delivery, understanding resistance mechanisms and minimizing off-target effects. Mn, an essential trace element, has been found to enhance T cell function and increase antitumor activity by activating the cGAS/STING pathway (49). Our study demonstrates that Mn can target cancer cells by degrading the oncoprotein GOLIM4, suggesting its potential as a targeted therapy. Nevertheless, further research is needed to evaluate treatment toxicity and identify optimal delivery routes before clinical implementation.

Several limitations of our study warrant discussion. First, we did not exhaustively investigate all 3q-encoded genes of interest, some of which may contribute to heightened secretion. Second, our short-term treatment studies did not assess the durability of the therapeutic response or the development of acquired resistance to Mn. Although no apparent signs of toxicity were observed, further preclinical studies are warranted to better evaluate the safety and efficacy of Mn treatment. Third, we did not investigate the combined effects of Mn with chemotherapy or immunotherapy that could enhance the clinical applications of Mn.

In conclusion, our study identified a chromosomal amplicon that serves as a platform for secretory regulation and underlies a targetable secretory addiction. These findings lay the groundwork for therapeutic approaches aimed at disrupting the secretory process in patients with cancer. The clinical relevance of 3q amplifications in cancer is underscored by their prevalence across multiple tumor types, the biological importance of a 3q-encoded functional circuitry that drives tumor progression, the availability of ddPCR assays that can identify patients with 3q-amplified tumors, and the potential efficacy of Mn-based treatments.

## Methods

### Sex as a biological variant.

Our study examined male mice because male animals exhibited less variability in phenotype.

### Human tumor studies.

ddPCR and quantitative reverse transcription PCR (qRT-PCR) assays were performed on a preexisting tissue bank of LUSC and PDX samples annotated on the basis of molecular and clinical parameters.

### Animal husbandry.

For subcutaneous tumor generation, nu/nu mice obtained from The Jackson Laboratory (*n* = 5–10 mice per group) were subcutaneously injected with 1 × 10^6^ human lung cancer cells. Orthotopic lung tumors were generated by intrathoracic injection of 1 × 10^6^ human lung cancer cells into nu/nu mice. Frozen LUSC PDX tissues obtained from an established tissue bank at MD Anderson Cancer Center were implanted subcutaneously into NOD.Cg-Prkdc^scid^ Il2rg^tm1Wjl^/SzJ mice (The Jackson Laboratory). The resulting fresh tumors were isolated, cut into small pieces (8–10 mm^3^), and transplanted into the flanks of nu/nu mice (*n* = 10 mice per group). Mice were treated daily with MnCl_2_ (50 mg/kg) or vehicle (PBS) via intraperitoneal injection for a period of 2 or 3 weeks. Tumor size and mouse body weight were monitored daily. Necropsies were performed to quantify primary tumor size and assess the numbers of distant metastases.

### Reagents.

We purchased SYBR Green, FBS, DMEM, RPMI Media 1640, Alexa Fluor–tagged secondary antibodies, Cell-Light Golgi-RFP, and DAPI from Life Technologies (Thermo Fisher Scientific); puromycin from InvivoGene; paraformaldehyde from Electron Microscopy Sciences; Transwell and Matrigel-coated Boyden chambers from BD Biosciences; G418 from Corning; CGP 54626 hydrochloride (HY-101378) and CGP7930 (HY-103502), EGTA (HY-D0861), BAPTA (HY-100168) from MedChemExpress; shRNAs against human GOLIM4 (TRCN0000143576 and TRCN0000140441) and human ATP2C1 (TRCN0000296815 and TRCN0000310147) and siRNAs against human GOLIM4 (SASI_Hs02_00345751 and SASI_Hs01_00148816), human ATP2C1 (SASI_Hs01_00149544 and SASI_Hs01_00044646), human CAB45 (SASI_Hs01_00117638 and SASI_Hs01_00117639), human GOLPH3 (SASI_Hs02_00355527 and SASI_Hs01_00133692), human APP (SASI_Hs01_00185801), human FAM3C (SASI_Hs01_00053865), human FBLN1 (SASI_Hs01_00209942), human GGH (SASI_Hs01_00166929), human HTRA1 (SASI_Hs01_00055644), human LAMC2 (SASI_Hs01_00136952), human LOXL2 (SASI_Hs01_00097658), human SERPINE2 (SASI_Hs01_00144800), human TIMP1 (SASI_Hs01_00019072), human PTX3 (SASI_Hs01_00211628), human GGA1 (SASI_Hs01_00063263), human BAG6 (SASI_Hs02_00319380), human UBL4A (SASI_Hs02_00345271), human GET3 (SASI_Hs01_00233482), human GET4 (SASI_Hs01_00153734), human TMED3 (SASI_Hs01_00024382), and siRNA Universal Negative Control number 2 (SIC002) from MilliporeSigma. We purchased primary antibodies against GOLIM4 (ALX-804-603-C100) from Enzo Life Sciences; GM130 (no. 560066) from BD Transduction Laboratories; α-tubulin (no. T9026) from MilliporeSigma; PARP-1 (no. 9542), cleaved caspase 3 (no. 9664), and His tag (no. 12698) from Cell Signaling Technology; Flag tag (F3165) and EGFP (G6539) from MilliporeSigma; hemagglutinin (HA tag) (nos. 3724 and 2367), β-actin (no. 4970), GM130 (no. 12480), and Golgin-97 (no. 13192) from Cell Signaling Technology; ATP2C1 (13310-1-AP) APP (22952-1-AP), GGH (18070-1-AP), LOXL2 (11405-1-AP), PTX3 (12306-1-AP), GGA1 (25674-1-AP), BAG6 (26417-1-AP), UBL4A (14253-1-AP), TMED3 (21902-1-AP), SPP1 (22952-1-AP), and CLU (12289-1-AP) from Proteintech; GABBR1 (ab55051) from Abcam; and VSV-G (IE9F9) from Kerafast. We purchased recombinant human APP (3466-PI-010) and PTX3 (10292-TS-050) from R&D Systems and recombinant human GGH (ab123172) from Abcam. Mn sensor M1 was synthesized by Ankona Datta’s laboratory (Tata Institute of Fundamental Research, Mumbai, India) and shared by this laboratory for the experiments (39).

### Cell lines.

Human lung cancer cell lines (A549, H1299, HCC15, H460, CALU-1, CALU-3, H358, H23, H441, H358, H596, H226, HCC95, H3255, H1819, and ABC1), human embryonic kidney 293T cells, immortalized human bronchial epithelial BEAS-2B cells, human breast cancer cell lines (MCF7, HCC70, and H1937), and human HN cancer cell lines (H157, FADU, and SCC25) were obtained from the American Type Culture Collection (ATCC). Murine lung cancer cell lines (307P, 393P, 344P, 344SQ, and 531LN2) were generated from tumors in *Kras^LA1/+^ Trp53^R172H/+^* mice as previously described (16). To isolate primary Thy-1^+^ cancer-associated fibroblasts (CAFs), freshly resected primary human LUADs were immediately perfused with PBS containing 2% FBS. The samples were mechanically minced using a macs dissociator, followed by enzymatic digestion at 37°C for 45 minutes with collagenase type I (3 mg/mL) and dispase II (4 mg/mL). The digested samples were then resuspended in PBS with 2% FBS to inactivate the enzyme. After filtration using 70 μm cell strainers and centrifugation at 400*g* at 4°C for 5 minutes, RBC lysis was performed using 1× RBC lysis solution for 1 minute at room temperature. The samples were then washed with PBS and 2% FBS to inactivate RBC lysis, followed by another centrifugation step. The cells were resuspended in PBS with 2% FBS and counted. Cell suspensions were incubated with the corresponding fluorochrome-conjugated mouse anti–human primary antibodies (CD45, Thy1/CD90, CD31, EPCAM; BD Biosciences) at a 1:100 dilution for 45 minutes on ice, with protection from light. After washing the cells in PBS with 2% FBS and centrifugation, compensation tubes were resuspended in 250 μL total volume, in the presence or absence of the viability dye 7-AAD. Flow cytometric sorting was then performed to isolate CD45^–^, CD31^–^, Ep-CAM^–^, and Thy-1^+^ cells in 1 mL PBS with 2% serum.

ABC1 cells, FADU cells, and CAFs were cultured in Eagle’s minimal essential medium supplemented with 10% FBS. SCC25 cells were cultured in DMEM/Ham’s F12 medium (Invitrogen, Thermo Fisher Scientific), supplemented with 1 mM sodium pyruvate, 1.1 mM hydrocortisone, and 10% FBS. BEAS-2B cells, 293T cells, and MCF-7 cells were cultured in DMEM containing 10% FBS. Human umbilical vein endothelial cells (HUVECs) were cultured in an EGM-2 Endothelial Cell Growth Medium-2 Bullet Kit (CC-5035, Lonza). All other human lung cancer cells and breast cancer cell lines were cultured in RPMI 1640 medium containing 10% FBS. Cells were maintained at 37°C in a humidified atmosphere with 5% CO_2_. Cell transfections were carried out using the jetPRIME Versatile DNA/siRNA transfection reagent (Polyplus). Stable cell transfectants were selected using puromycin (for pLKO.1 vectors) or G418 (for pcDNA3.1 and pEGFP-C3 vectors). GOLIM4-KO H1299 cells were generated using the CRISPR/Cas9 system in the Cell-Based Assay Screening Service Core Facility at Baylor College of Medicine as previously described (10). Two guide RNAs (gRNA-1: 5′-ATCTTTGCAGAGCCAACACG-3′; gRNA-2: 5**′**-CAAGAACTTTCTAAGCTAAA-3′) were used. GOLIM4-KO clones were confirmed by WB analysis. *ATP2C1*-KO H1299 and 344SQ GOLIM4–KO cells were generated using the CRISPR/Cas9 method as previously reported (50). Briefly, for *ATP2C1* KO in H1299 cells, 2 sgRNA sequence were selected from the GenScript’s Broad sgRNA Database and inserted into the lentiCRISPR v2 vector (gRNA-1: 5′-ATGCTTGCAACTTCACTGACTGG-3′; gRNA-2: 5′-AATATCCTCTCCATGCAATTAGG-3′). To generate *Golim4*-KO 344SQ cells, 2 sgRNAs (gRNA-1: 5′-CACCGGTGCTTCTAACTTATAAACA-3′; gRNA-2: 5′-CACCGGCACAAGAAACACTTAACAA-3′) were inserted into the lentiCRISPR v2 vector. H1299 and 344SQ cells were transfected with the lentiCRISPR v2-ATP2C1/Golim4 sgRNA using jetPRIME transfection reagent. Forty-eight hours after transfection, cells were selected with puromycin for 2 days, and the resulting cells were subjected to limiting dilution plating in 96-well plates for single clone isolation. The candidate clones were confirmed by WB analysis using an anti-ATP2C1 or anti-GOLIM4 antibody.

### Vector construction.

The human GOLIM4 coding sequences were isolated by performing PCR on cDNA prepared from H1299 cells and then cloned into pcDNA3.1(-) (Invitrogen, Thermo Fisher Scientific). Truncations and mutations were generated by PCR. To generate the TurboID-GOLIM4 construct, GOLIM4 coding sequences were inserted into the Flag-TurboID-pcDNA3 vector using XhoI and XbaI. The primers used are listed in Supplemental Table 4.

The EGFP-VSV-G (ts045) expression construct (Addgene plasmid 11912) was a gift from Jennifer Lippincott-Schwartz (Janelia Research Campus, Howard Hughes Medical Institute, Ashburn, Virginia, USA). ATP2C1-HA-pLPCX and APT2C1-D350A-HA-pLPCX expression constructs were gifts from Julia von Blume (Yale School of Medicine, New Haven, Connecticut, USA). EGFP-RAB6A (Addgene plasmid 49469) was a gift from Marci Scidmore (Cornell College of Veterinary Medicine, Tompkins County, New York, USA). pEGFP-n1-APP (Addgene plasmid 69924) was a gift from Zita Balklava and Thomas Wassmer (Aston University, Aston Triangle, Birmingham, United Kingdom). pcDNA3.tgoGAP1 (Addgene plasmid 78737) was a gift from Teresa Alonso (Hospital Universitario Ramón y Cajal, Madrid, Spain). GFP-GGA1 (Addgene plasmid 178459) was a gift from Juan Bonifacino (Eunice Kennedy Shriver National Institute of Child Health and Human Development, NIH, Bethesda, Maryland, USA). lentiCRISPR v2 (Addgene plasmid 52961) was a gift from Feng Zhang (Massachusetts Institute of Technology, Cambridge, Massachusetts, USA).

### Cell proliferation, colony formation, apoptosis, migration, and invasion assays.

Cell proliferation assays were conducted using Cell Proliferation Reagent WST-1 (Roche), following the manufacturer’s instructions. Colony formation assays at low density on plastic and in soft agarose were performed as previously described (51). Flow cytometric analysis of apoptotic cells was carried out using the Dead Cell Apoptosis Kit (Thermo Fisher Scientific, V13242), following the manufacturer’s recommended protocol. Migration and invasion assays were performed using Transwell and Matrigel-coated Boyden chambers, respectively, as previously described (11). HUVEC and CAF recruitment assays were conducted by seeding 2 × 10^4^ HUVECs or 10^4^ CAFs into the top chambers and H1299 cells into the bottom chambers of Transwell plates. After 12 hours, the migrated HUVECs or CAFs were then stained with 0.1% crystal violet, photographed, and counted.

### WB analysis and immunoprecipitation assays.

WB analysis was performed as previously described (11). For immunoprecipitation, H1299 cells were transfected with the indicated expression vectors, lysed after 48 hours in 1× RIPA buffer (Cell Signaling Technology), and incubated with antibodies at 4°C overnight. The immune complex was captured with protein G agarose beads (Cell Signaling Technology), washed with 1× RIPA buffer once and 1× PBS 3 times, and boiled in 1× sodium dodecyl sulfate loading buffer at 98°C for 10 minutes. The resulting samples were subjected to WB analysis.

### RNA-Seq.

Triplicate samples of total RNA were obtained from H520 cells transfected with either an siRNA against GOLIM4 or a control siRNA. RNA-Seq was performed by the MD Anderson Illumina Next-Generation Sequencing Core using the NovaSeq 6000 whole transcriptome sequencing protocol. The resulting RNA-Seq fastq reads were mapped to the human reference genome GRCh38 (hg38) and the transcriptome gene annotation GENCODE V31 using RSEM version 1.3.3. Gene expression levels were estimated on the basis of normalized read counts in tags per million reads (TPM). The gene count data matrix was logarithmically transformed and normalized using EdgeR version 3.26.8 with default settings. Differentially expressed genes between the siGOLIM4 and control groups were identified using a FDR cutoff of 0.05 or lower and an absolute log_2_ fold change 1 or higher. The raw data for this study have been deposited in the NCBI’s Gene Expression Omnibus (GEO) database (GEO GSE237935).

### Mn-pulldown assay.

H1299 cells (2 × 10^6^) were pretreated with 1 mM MnCl_2_ for 2 hours and lysed in 1 mL ice-cold RIPA buffer for 15 minutes on ice. For affinity purification, 80 μL uncharged Profinity IMAC resins (1560121, Bio-Rad) were equilibrated with sodium phosphate buffer (pH 8.0) and then incubated with the total cell lysates overnight at 4°C. After washing the resins 4 times with PBS, the eluted protein was subjected to LC-MS analysis or denatured in 5× SDS loading buffer at 98°C for 10 minutes for WB analysis.

### qRT-PCR analysis.

To isolate total RNA from cells, we used the RNeasy Mini Kit (QIAGEN). Reverse transcription was carried out using qScript cDNA SuperMix (Quanta Biosciences). Genomic DNA was extracted from cells using DNeasy Blood and Tissue Kits (QIAGEN). Gene copy numbers and mRNA levels were assessed using SYBR Green Real-Time PCR Master Mixes (Thermo Fisher Scientific) and normalized to ribosomal protein L32 (Rpl32) mRNA. The specific PCR primers used in this study are listed in Supplemental Table 1.

### ddPCR.

GOLIM4 (5′-FAM-CACCAAGACATACATACAC-BGQ-3′) and RPP30 (HEX) probes (Bio-Rad) were diluted in Bio-Rad ddPCR SuperMix and mixed with 4.4 units of HindIII restriction enzyme diluted in NEB buffer 2.1 (New England BioLabs) to make a mastermix. A total of 10 ng of each sample was added to the master mix in a 96-well plate. Each sample was run in duplicate. The droplets were automatically generated using the Auto-DG (Bio-Rad), after which they were amplified in a deep-well thermocycler. The droplets were detected using a QX 200 droplet reader (Bio-Rad) and analyzed with QuantaSoft (Bio-Rad). The GOLIM4 copy number (normalized to RPP30) of each sample was determined on the basis of the ratio of normalized PCR values.

### CM sample preparation and transfer.

Following a previously described protocol (52), CM samples were isolated, filtered using a 0.45 μm filter, and combined with an equal volume of complete growth medium, resulting in a final concentration of 5% FBS. This mixture was then applied to cells. For the cell proliferation and colony formation assays, CM samples were replaced every 2 days.

### LC-MS analysis.

H1299 cells (2 × 10^6^) were seeded in a 10 cm plate, and serum-free medium was added 24 hours later. CM samples were collected after 16 hours, filtered, and concentrated using Amicon Ultra-15 10K and Ultra 0.5 10K centrifugal filters. To solubilize the samples, 25 μL 5% SDS and 50 mM triethylammonium bicarbonate (TEAB) (pH 7.55) were added. The solution was centrifuged at 17,000*g* for 10 minutes to remove debris. Proteins were reduced by adding 20 mM Tris(2-carboxyethyl)phosphine (TCEP) (Thermo Fisher Scientific, 77720) and incubated at 65°C for 30 minutes. After cooling to room temperature, 1 μL of 0.5 M iodoacetamide was added, and the solution was allowed to react in the dark for 20 minutes. Then, 2.75 μL of 12% phosphoric acid was added, followed by the addition of 165 μL binding buffer (90% methanol, 100 mM TEAB, final pH 7.1). The resulting solution was passed through an S-Trap spin column (protifi.com) using a benchtop centrifuge (30-second spin at 4,000*g*). The spin column was washed 3 times with 400 μL binding buffer. Trypsin was added to the protein mixture at a ratio of 1:25 in 50 mM TEAB (pH 8), and the solution was incubated at 37°C for 4 hours. Peptides were eluted with 80 μL 50 mM TEAB, followed by 80 μL 0.2% formic acid, and finally 80 μL 50% acetonitrile, 0.2% formic acid. The combined peptide solution was dried using a speed vac and then resuspended in an autosampler vial with 2% acetonitrile, 0.1% formic acid, and 97.9% water for LC-MS analysis (10). For all proteomics analysis, samples were provided as 3 bioreplicates.

In order to identify proteins that interact with GOLIM4, a TurboID-GOLIM4 construct was introduced into H1299 cells. After culturing in medium supplemented with or without 100 μM biotin for 1 hour, cell lysates were collected, and biotinylated proteins were purified using Pierce streptavidin agarose beads (Thermo Fisher Scientific). The proteins bound to the beads were then identified through LC-MS analysis, following the established protocol (52).

### Secretory vesicle trafficking assays.

To assess secretory vesicle biogenesis and trafficking, RAB6A-EGFP reporter assays and VSV-G trafficking assays, respectively, were performed, as previously described (52). For RAB6A-EGFP assays, live-cell imaging was conducted on RAB6A-EGFP–transfected H1299 cells, and extra-Golgi RAB6A^+^ vesicles were quantified using ImageJ software (NIH). For VSV-G assays, cells were transiently transfected with EGFP-VSV-G (ts045) and subjected to a temperature shift from permissive (32°C) to restrictive (40°C) temperatures for 20 hours. Subsequently, the cells were transferred back to the permissive temperature of 32°C for 1 hour in the presence of 100 μg/mL cycloheximide. After fixation, exofacial and total VSV-G were detected in nonpermeabilized cells using an anti–VSV-G antibody and by measuring EGFP signal intensity, respectively. The trafficking of VSV-G to the plasma membrane was quantified on the basis of a ratio of fluorescence signal from exofacial (surface) VSV-G to EGFP (total) signal intensity.

To quantify secretory cargo exit from the Golgi, H1299 cells were transiently transfected with an EGFP-tagged APP expression vector. After 24 hours, the cells were incubated at a restrictive temperature of 21.5°C for 2 hours in RPMI supplemented with 0.2% FBS and 100 μg/mL cycloheximide. Subsequently, the cells were transferred to a permissive temperature of 37°C and fixed at predetermined time points. Cells were permeabilized and subjected to staining with antibodies against Golgin-97 and DAPI. The APP^+^ extra-Golgi puncta were counted and quantified using ImageJ.

### Intra-Golgi Mn and Ca^2+^ assays.

For intra-Golgi Mn assays, H1299 cells were transduced with CellLight Golgi-RFP (Thermo Fisher Scientific) for 24 hours and exposed to specified concentrations of MnCl_2_ for 1 hour before being treated with 5 μM Mn sensor M1 for 15 minutes. The fluorescence intensity of the M1 sensor was quantified by averaging the measured intensity within the Golgi marker region as previously reported (39). For intra-Golgi Ca^2+^ assays, H1299 cells were transfected with the Ca^2+^ reporter pcDNA3.tgoGAP1 and CellLight Golgi-RFP and then treated with Ca^2+^ chelators EGTA (1 mM) and BAPTA (10 μM) for 4 hours or an siRNAs against the Golgi Ca^2+^ transporter ATP2C1 for 48 hours. Live-cell imaging was conducted using a confocal microscope. The fluorescence intensity of the Ca^2+^ sensor was quantified and normalized to Golgi-RFP signals.

### Microscopy and image analysis.

Cells were imaged using an Eclipse Ti inverted microscope with an A1+ confocal scanner (Nikon, Japan), equipped with diode lasers of 405, 488, 561, and 640 nm wavelengths, high-sensitivity Gallium arsenide phosphide and photomultiplier tube detectors, and either a ×60 1.4 NA Oil or a ×100 1.45 NA Oil objective. NIS-Elements software (Nikon), version 4.40 (Build 1084), was utilized for image acquisition. For high-resolution imaging, *Z*-stacks were acquired sequentially at slow scan speed using a frame size of 512 × 512 or 1,024 × 1,024, low pinhole, and optimized detector gain. Nyquist sampling criteria were followed, and laser power was adjusted to minimize bleaching. After acquisition, images were processed and deconvolved using Huygens Professional, version 18.04 (Scientific Volume Imaging, Netherlands), with the Classic Maximum Likelihood Estimation algorithm. Image analysis was performed using Fiji software (ImageJ, version 1.51s), Huygens Professional, or NIS Elements. Immunofluorescence procedures were carried out following previously described methods (12).

### Immunohistochemical analysis of tumor tissues.

Tissue sections of 4 μm thickness from formalin-fixed, paraffin-embedded lung tissues were stained using the Leica Bond Max automated stainer (Leica Biosystems) automated staining platform. Following the Leica Bond protocol, the tissue sections were deparaffinized and rehydrated. Antigen retrieval was performed using Bond Solution no. 2 (Leica Biosystems, equivalent EDTA, pH 9.0) for 30 minutes. Primary antibodies (CD31, dilution 1:100, Cell Signaling Technology, 77699; αSMA, dilution 1:300, Abcam, ab5694; PCNA, dilution 1:200, Cell Signaling Technology, 13110) were incubated for 15 minutes at room temperature. The primary antibody was detected using the Bond Polymer Refine Detection kit (Leica Biosystems) with DAB as the chromogen. Slides were counterstained with hematoxylin, dehydrated, and coverslipped. Immunostained sections were digitally scanned using the Aperio AT2 slide scanner (Leica Biosystems) under ×20 objective magnification. Digital image analysis was performed using pathologist-trained specific algorithms for quantification.

### Statistics.

Unless stated otherwise, the results shown are representative of replicated experiments and are the mean ± SDs from triplicate samples or randomly chosen cells within a field. Statistical evaluations were carried out with GraphPad Prism 6 (GraphPad Software). An unpaired, 2-tailed Stuof secretory vesicle biogedent *t* test was used to compare the mean values of 2 groups. ANOVA with Dunnett’s test was used for comparison of multiple treatments with a control. *P* values of less than 0.05 were considered statistically significant. Kaplan-Meier survival data were generated using GEPIA2 (53) or cBioportal (54–56). Plots were generated for the respective groups using GraphPad Prism, version 9.

### Study approval.

Human tumor specimens were obtained through an IRB-approved protocol and with informed consent, and the analysis of the human tissue specimens was approved by an IRB at MD Anderson Cancer Center. All mouse studies were conducted in accordance with the guidelines and regulations approved by the IACUC at The University of Texas MD Anderson Cancer Center. Mice received standard care and were euthanized according to the established protocols of the IACUC.

### Data availability.

The RNA-Seq data are available in the NCBI’s GEO database (GEO GSE237935). All other data associated with this study are present in the manuscript or in the supplemental materials and are available in the Supporting Data Values file.

## Author contributions

XT and SW conceived, designed, executed, and interpreted the molecular biology, cell biology, and in vivo experiments. GYX conceived, designed, executed and interpreted the VSV-G trafficking experiments. CW conceived, designed, and executed the *GOLIM4* and *ATP2C1*-KO cell experiments. XL assisted XT with the in vivo experiments. BZ assisted XT with cell culturing and WB assays. YJ bred the mice for the in vivo studies. DYD conceived, designed, executed, and interpreted the ddPCR assay. YX conceived and executed the RNA-Seq data analysis. JW directed YX on data analysis. KG synthesized the Mn sensor M1 and provided guidance on the Mn sensor experiment. AP maintained and provided the LUSC PDX. MPK maintained and provided the pancreatic ductal adenocarcinoma PDX. FC performed TCGA copy number analysis. CJC directed and interpreted TCGA copy number analysis. WKR directed and interpreted the mass spectrometry experiments. JAR and MPK maintained and provided the pancreatic ductal adenocarcinoma PDX. JMK conceived and supervised the project and contributed to the design and interpretation of all experiments.

## Supplementary Material

Supplemental data

Unedited blot and gel images

Supplemental table 1

Supplemental table 2

Supplemental table 3

Supplemental table 4

Supporting data values

## Version 1 04/25/2024

In-Press Preview

## Version 2 06/17/2024

Electronic publication
