# Supplementary material for: Chromosomal 3q amplicon encodes essential regulators of secretory vesicles that drive secretory addiction in cancer
Source: J Clin Invest. 2024 Apr 25;134(12):e176355. doi: 10.1172/JCI176355 (PMC11178546; doi:10.1172/JCI176355)
Supplement: Supplemental data [file jci-134-176355-s234.pdf]

Supplementary figures and legends

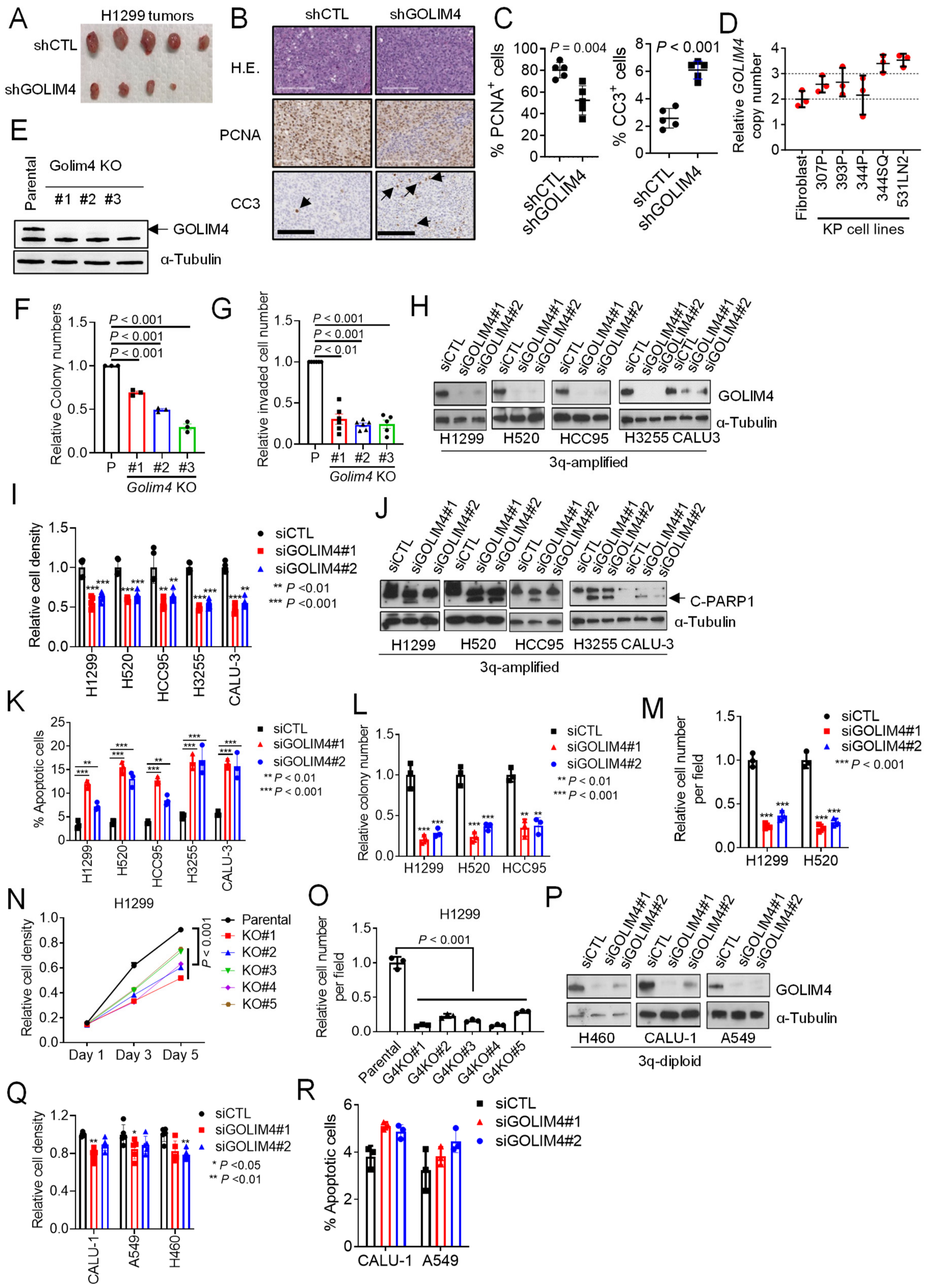

Figure S1. GOLIM4 is a pro-tumorigenic effector in 3q-amplified lung cancer. (A) Images of subcutaneous tumors generated by shRNA-transfected H1299 cells. (B) PCNA- and cleaved caspase-3 (CC3)-positive cells (arrows) detected by immunohistochemical staining of tumor sections from (A). Hematoxylin and eosin staining (H.E.). (C) Quantification of PCNA (left plot) or CC3 (right plot) positive cells from (B). (D)qPCR analysis of GOLIM4 copy number in murine lung cancer cell lines. (E) WB analysis of GOLIM4 protein levels in parental or GOLIM4 KO 344SQ cells. Arrow indicates the GOLIM4 band. (F and G) Colony formation (F) and Boyden chamber transwell invasion (G) assays on cells in (E). (H) WB analysis of 3q-amplified lung cancer cells transfected with indicated siRNAs. (I) Relative cell density in monolayer culture determined by WST-1 assays. (J and K) Detection of apoptotic cells by WB analysis of cleaved PARP1 (CPARP1) (J) and Annexin V/propidium iodide (PI) flow cytometry (K). The tubulin blots for H3255 and CALU-3 cells in panels H and J are from the same experiment. (L) Relative colony numbers in soft agarose. (M) Boyden chamber migration assay. (N and O) Relative cell density assays (N) and Boyden chamber migration assays (O) on parental and GOLIM4 knockout (KO) H1299 cells. (P) WB confirmation of target gene depletion in 3q-diploid lung cancer cells transfected with GOLIM4 siRNAs. (Q and R) Relative cell density assays (Q) and Annexin V/PI flow cytometry apoptosis assays (R) on cells in (P). Data indicate the mean ± SD from a single experiment incorporating biological replicate samples (n = 3, unless otherwise indicated) and are representative of at least 2 independent experiments. P values were determined using two-tailed Student's t test (for C) or one-way ANOVA test (for F, G, I, K-O, Q, and R).

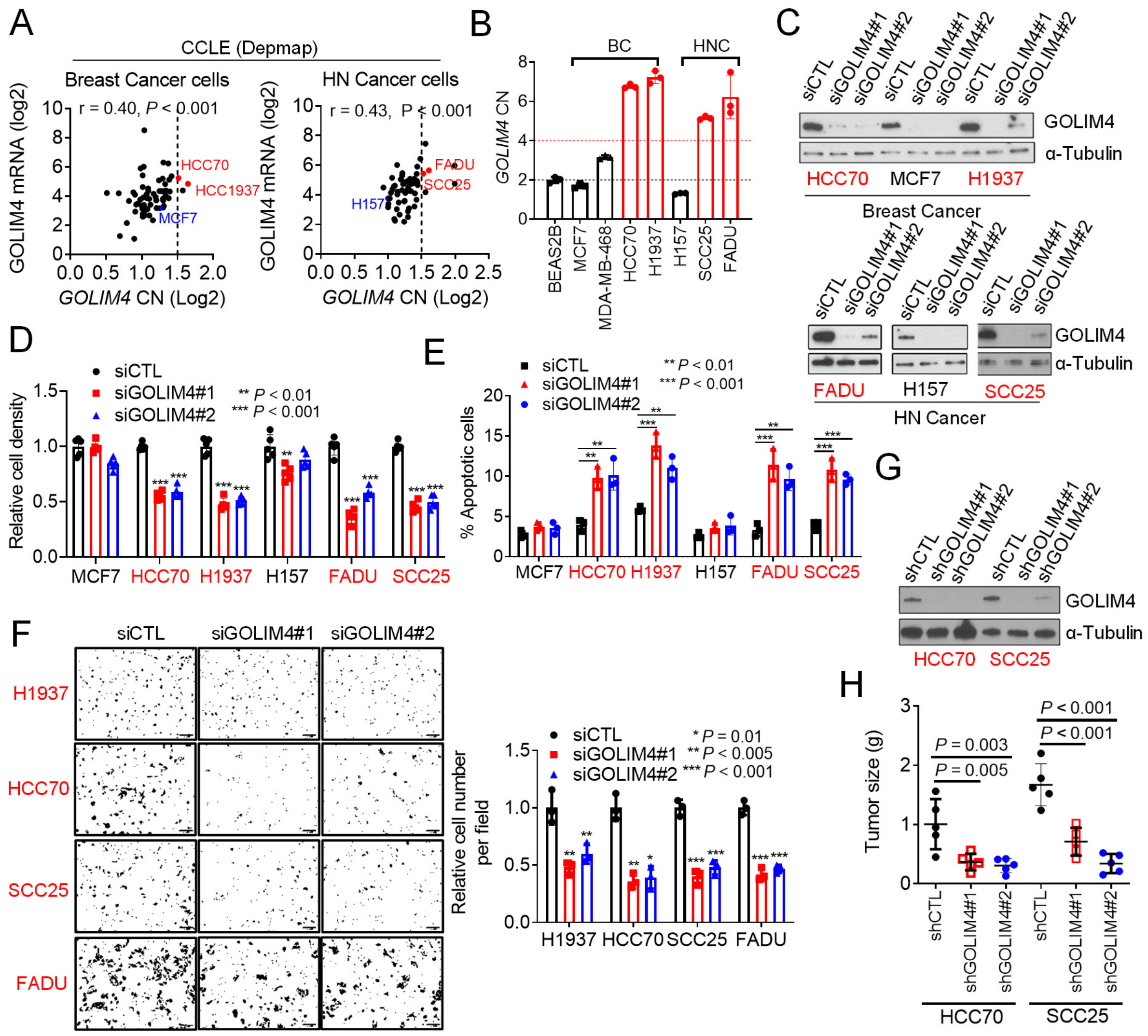

Figure S2. GOLIM4 is a pro-tumorigenic effector in 3q-amplified breast and head and neck (HN) cancers. (A, B) Correlation of *GOLIM4* copy numbers and mRNA levels in CCLE database (http:depmap.org) (A). qPCR analysis of *GOLIM4* CN in breast and HN cancer cell lines (B). 3q-diploid (black) and -amplified (red). (C) WB confirmation of target gene depletion in siRNA-transfected breast and HN cancer cell lines. 3q-amplified (red) or -diploid (black). (D-F) Relative cell density assays (D), Annexin V/PI flow cytometric apoptosis assays (E), and Boyden chamber migration assays (F) on cells generated in (C). Migrated cells were imaged and quantified (graph). (G) WB analysis of shRNA-transfected HCC70 and SCC25 cells. (H) Flank tumors generated by cells in (G). Data indicate the mean ± SD from a single experiment incorporating biological replicate samples (n = 3, unless otherwise indicated) and are representative of at least 2 independent experiments. P values were determined using one-way ANOVA test (for D, E, F, and H).

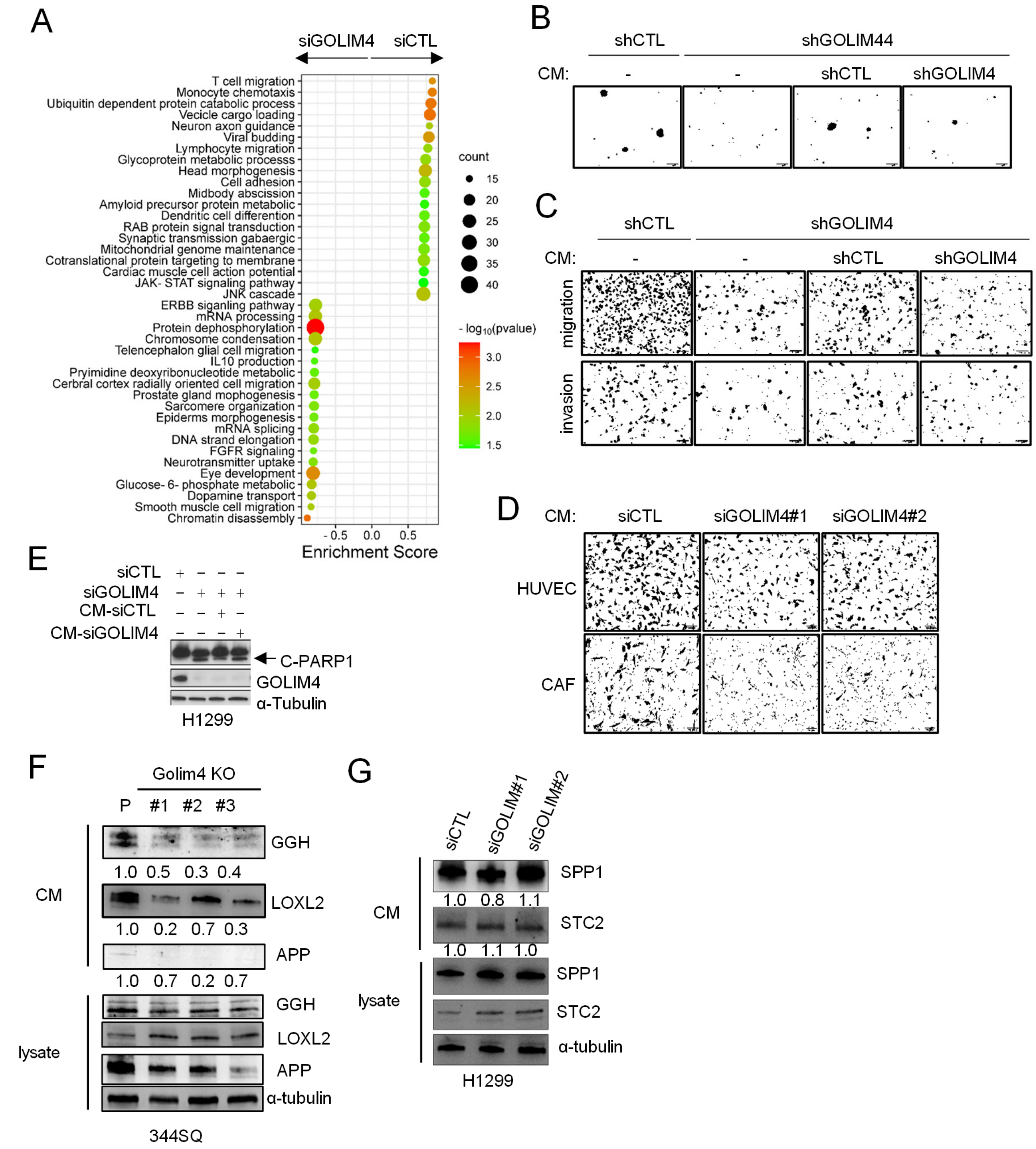

Figure S3. GOLIM4-dependent secretory functions. (A) Gene ontology analysis of differentially expressed genes in GOLIM4-deficient (siGOLIM4) or -replete (siCTL) H520 cells. (B) Images of colonies formed in soft agar by shRNA-transfected H1299 cells following treatment with CM samples from shRNA-transfected H1299 cells. (C) Images of migrated and invaded H1299 cells. (D) Images of migrated HUVECs and CAFs. Bottom wells of Boyden chambers loaded with CM samples from siRNA-transfected H1299 cells. (E) WB analysis of cleaved PARP1 (C-PARP1) in siRNA-transfected H1299 cells treated with CM samples from siRNA-transfected H1299 cells. GOLIM4 and tubulin included as controls. (F and G) WB analysis of indicated protein levels in the CM and lysates from 344SQ (F) or H1299 (G) cells.

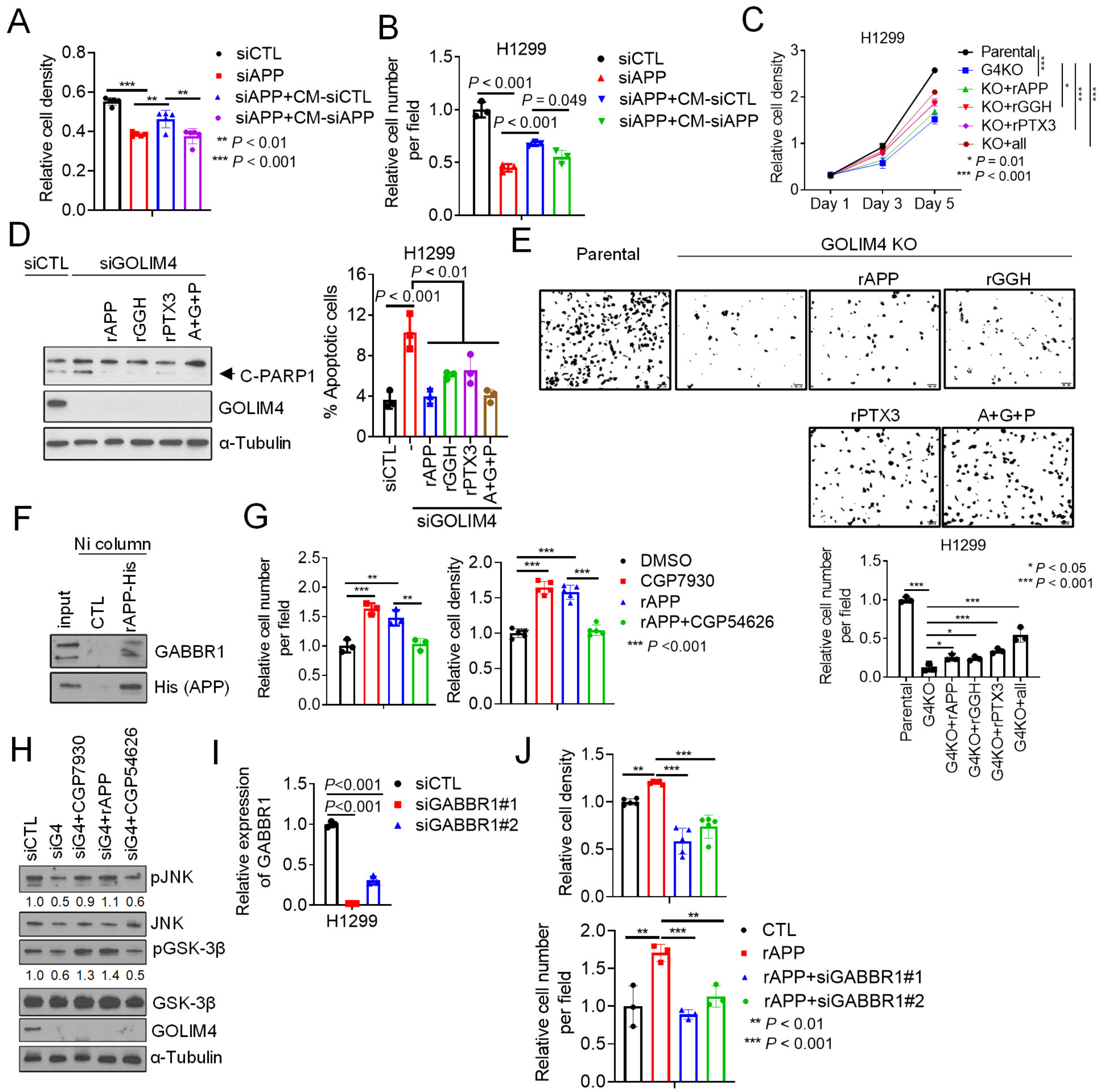

Figure S4. Pro-survival signals activated by an APP-dependent autocrine loop. (A and B) Relative cell density assays (A) and Boyden chamber migration assays (B) on APP-deficient H1299 cells reconstituted with CM samples from siRNA-transfected H1299 cells. (C) Relative density of parental and *GOLIM4* KO (G4KO) H1299 cells in the presence or absence of recombinant secreted proteins. (D) Detection of apoptotic cells by WB analysis of C-PARP1 (gel) or annexin V/PI flow cytometric analysis (graph). (E) Boyden chamber migration assay on *GOLIM4* KO cells treated with recombinant secreted proteins. (F) Nickle resin pull-down assay to detect APP-binding activity in H1299 cells. H1299 cells were treated with recombinant His-tagged APP protein (rAPP-His). After incubating lysates on Ni resin, bound proteins were eluted and subjected to WB analysis to detect the APP receptor GABBR1. (G) Boyden chamber migration assays (left) and relative cell density assays (right) on H1299 cells treated with recombinant APP and GABBR1 antagonist (CGP54626) or agonist (CPG7930). (H) WB analysis of total JNK, phosphorylated (p-) JNK (pJNK), GSK-3β, and p-GSK-3β levels in siRNA-transfected H1299 cells treated with

recombinant APP and CGP54626. Relative densitometric values are shown under the gel lanes. α-Tubulin as loading control. (I) qPCR confirmation of target gene depletion in siRNA-transfected H1299 cells. (J) Relative cell density assays (top) and Boyden chamber migration assays (bottom) on siRNA-transfected *GOLIM4* KO H1299 cells treated with recombinant APP. Data indicate the mean ± SD from a single experiment incorporating biological replicate samples (n = 3, unless otherwise indicated) and are representative of at least 2 independent experiments. P values were determined using one-way ANOVA test (for A, B, C, D, E, G, I and J).

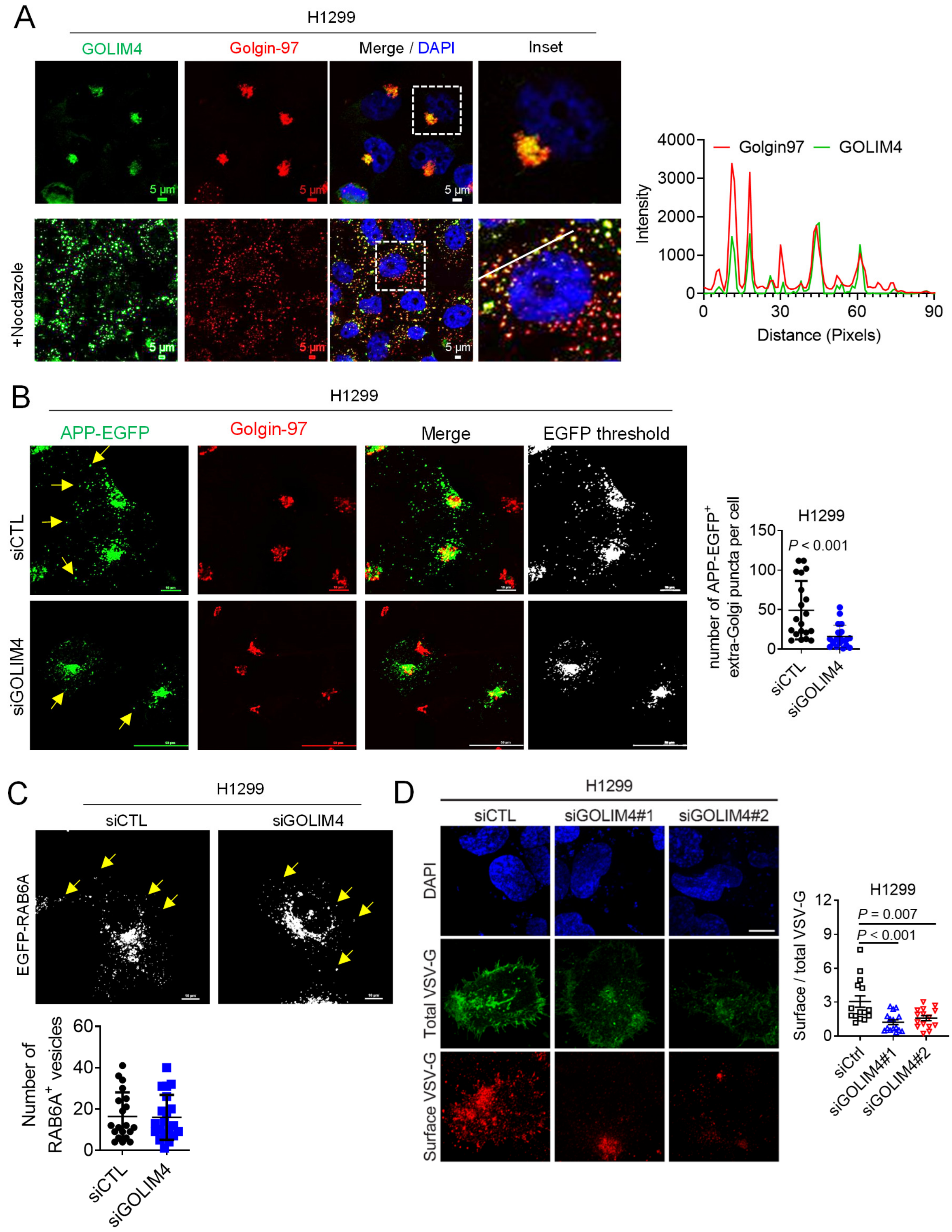

Figure S5. GOLIM4 regulates secretory vesicle biogenesis. (A) Confocal micrographs of H1299 cells demonstrates Golgi localization of GOLIM4. Cells were treated with nocodazole to disperse the Golgi, fixed, and co-stained with anti-GOLGIN-97 and anti-GOLIM4 antibodies. Scale bar: 5 μm. GOLGIN-97 and GOLIM4 signal intensities (Y-axis) plotted on a line drawn from plasma membrane inwards (X-axis) (line graph). (B) Single and merged confocal micrographs of APP-

EGFP in siRNA-transfected H1299 cells. Golgi marker (anti-Golgin-97). APP-EGFP-positive vesicles (arrows). Scale bar: 50 μm. APP-EGFP-positive vesicles per cell (dot) (graph). (C) Confocal micrographs of RAB6A+ vesicles (yellow arrows). Scale bar: 10 μm. Results were quantified per cell (dot plots). (D) Single-channel and merged confocal micrographs of total and surface EGFP-tagged temperature-sensitive-mutant vesicular stomatitis virus-G (VSV-G) in siRNA-transfected H1299 cells. EGFP–VSV-G was imaged 30 minutes after transfer to the permissive temperature. Plot shows the ratio of surface VSV-G to total VSV-G in each cell (dot). Scale bar: 20 μm. Data indicate the mean ± SD from a single experiment incorporating biological replicate samples (n = 3, unless otherwise indicated) and are representative of at least 2 independent experiments. P values were determined using two-tailed student's t test (for B and C) or one-way ANOVA test (for D).

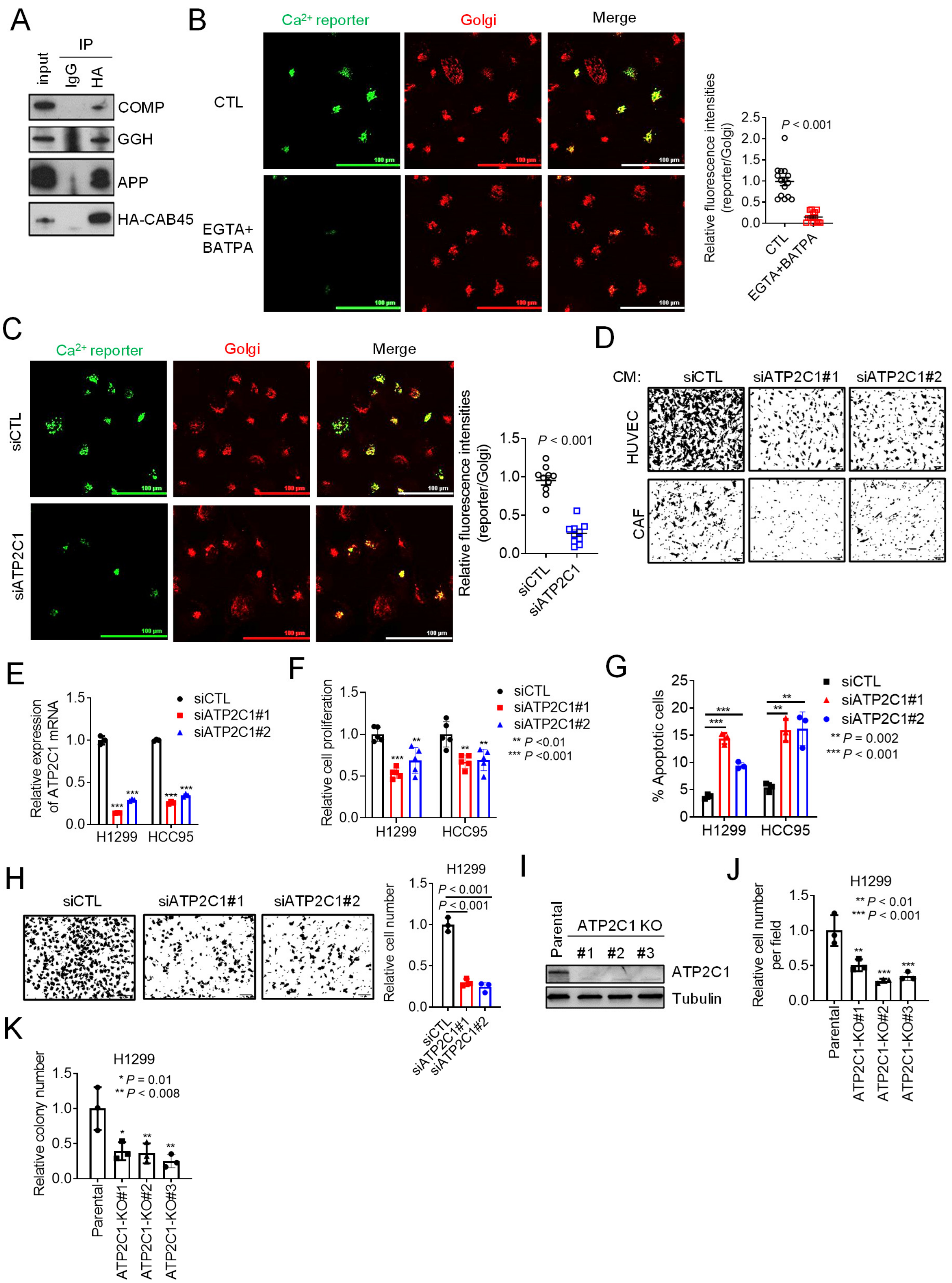

Figure S6. Secretion is ATP2C1-dependent. (A) IP/WB analysis detects CAB45 in complex with GOLIM4-dependent secreted proteins. The CAB45-interacting protein COMP included as a positive control. (B and C) Single channel and merged confocal micrographs of a Golgi-localized

calcium (Ca) sensor (green) as a readout of intra-Golgi Ca levels in H1299 cells. Signal specificity confirmed by Ca chelator treatment (B) or ATP2C1 siRNA transfection (C). CellLight Golgi-RFP (red). Relative intensity of Ca reporter per cell (dot) (graph). Scale bar: 100 µm. (D) Images of migrated HUVECs and CAFs in Boyden chambers. Bottom wells loaded with CM samples as chemoattractants. Scale bar: 100 µm. (E) qPCR confirmation of target gene depletion in siRNA-transfected H1299 cells and HCC95 cells. (F and G) Relative cell density assays (F) and Annexin V/PI flow cytometric apoptosis assays (G) on cells in (E). (H) Boyden chamber migration assay on siRNA-transfected H1299 cells. (I) WB confirmation of target gene depletion in *ATP2C1* KO H1299 cells. (J and K) Boyden chamber migration assays (J) and soft agar colony formation assays (K) on cells in (I). Data indicate the mean ± SD from a single experiment incorporating biological replicate samples (n = 3, unless otherwise indicated) and are representative of at least 2 independent experiments. P values were determined using two-tailed student's t test (for B and C) or one-way ANOVA test (for E-G, H, J, and K).

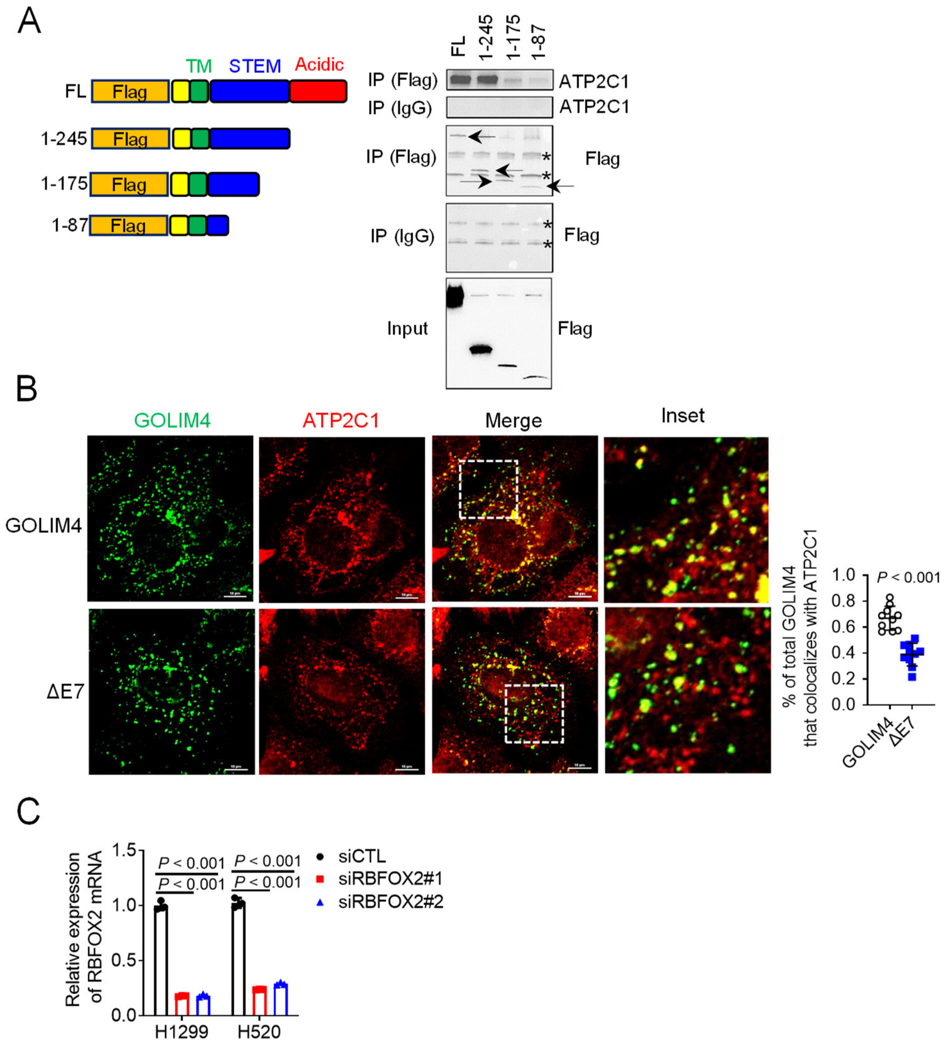

Figure S7. ATP2C1-binding activity localizes to an alternatively spliced exon in GOLIM4. (A) Full length (FL) and truncated GOLIM4 protein expression constructs. IP/WB analysis of H1299 cells transfected with indicated GOLIM4 constructs (gel). GOLIM4 (arrowheads) and non-specific

bands (asterisks). (B) Single-channel and merged confocal micrographs of *GOLIM4* KO cells reconstituted with FL or ∆E7 GOLIM4. Cells co-stained with anti-GOLIM4 and anti-ATP2C1 antibodies. Scatter plot shows the percentages of GOLIM4 that co-localizes with ATP2C1 in each cell (dot). Scale bar: 10 µm. (C) qPCR confirmation of target gene depletion in siRNA-transfected H1299 cells and H520 cells. Data indicate the mean ± SD from a single experiment incorporating biological replicate samples (n = 3, unless otherwise indicated) and are representative of at least 2 independent experiments. P values were determined using two-tailed student’s t test (for B) or one-way ANOVA test (for C).

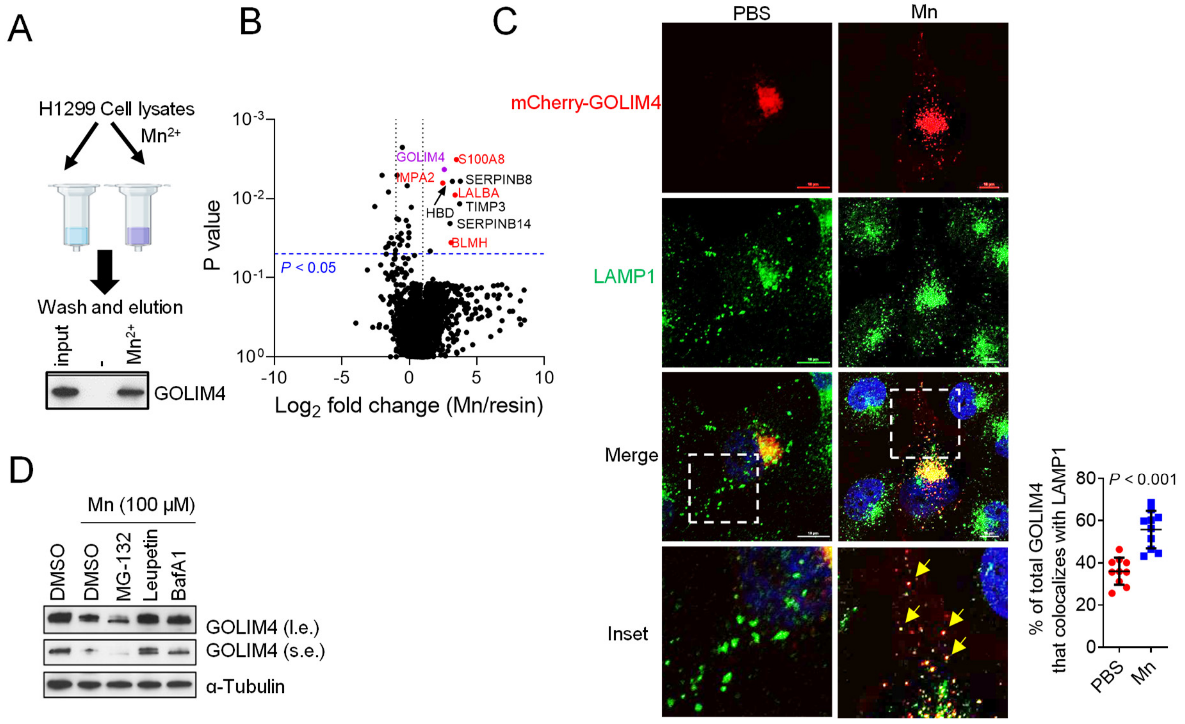

Figure S8. Mn-binding induces GOLIM4 translocation to lysosomes for degradation. (A) Mn pull-down assay. Following Mn treatment, H1299 cells were lysed and incubated with IMAC resin. GOLIM4 detected by WB analysis of eluted proteins. (B) Volcano plot illustration of Mn-binding proteins identified by LC-MS analysis. Results expressed as a log2 ratio (Mn/resin). Y-axis: p values; X-axis: fold-change. GOLIM4 (purple) and other known Mn-binding proteins (red) are indicated. (C) Single-channel and merged confocal micrographs detect increased co-localization of GOLIM4 and LAMP1 in Mn-treated H1299 cells. Cells transfected with mCherry-tagged GOLIM4 and stained with anti-LAMP1 antibody. Lysosomal GOLIM4 (arrows) in inset (2.5x higher magnification). Scale bar: 10 µm. Scatter plot shows the percentages of GOLIM4 that colocalize with LAMP1 in each cell (dot). (D) WB analysis of GOLIM4 levels in H1299 cells co-treated with Mn and proteasome (MG-132) or lysosome inhibitors (Leupeptin and BafA1). Long exposure (l.e.). Short exposure (s.e.). Data indicate the mean ± SD from a single experiment incorporating biological replicate samples (n = 3, unless otherwise indicated) and are representative of at least 2 independent experiments. P values were determined using two-tailed student’s t test (for C).

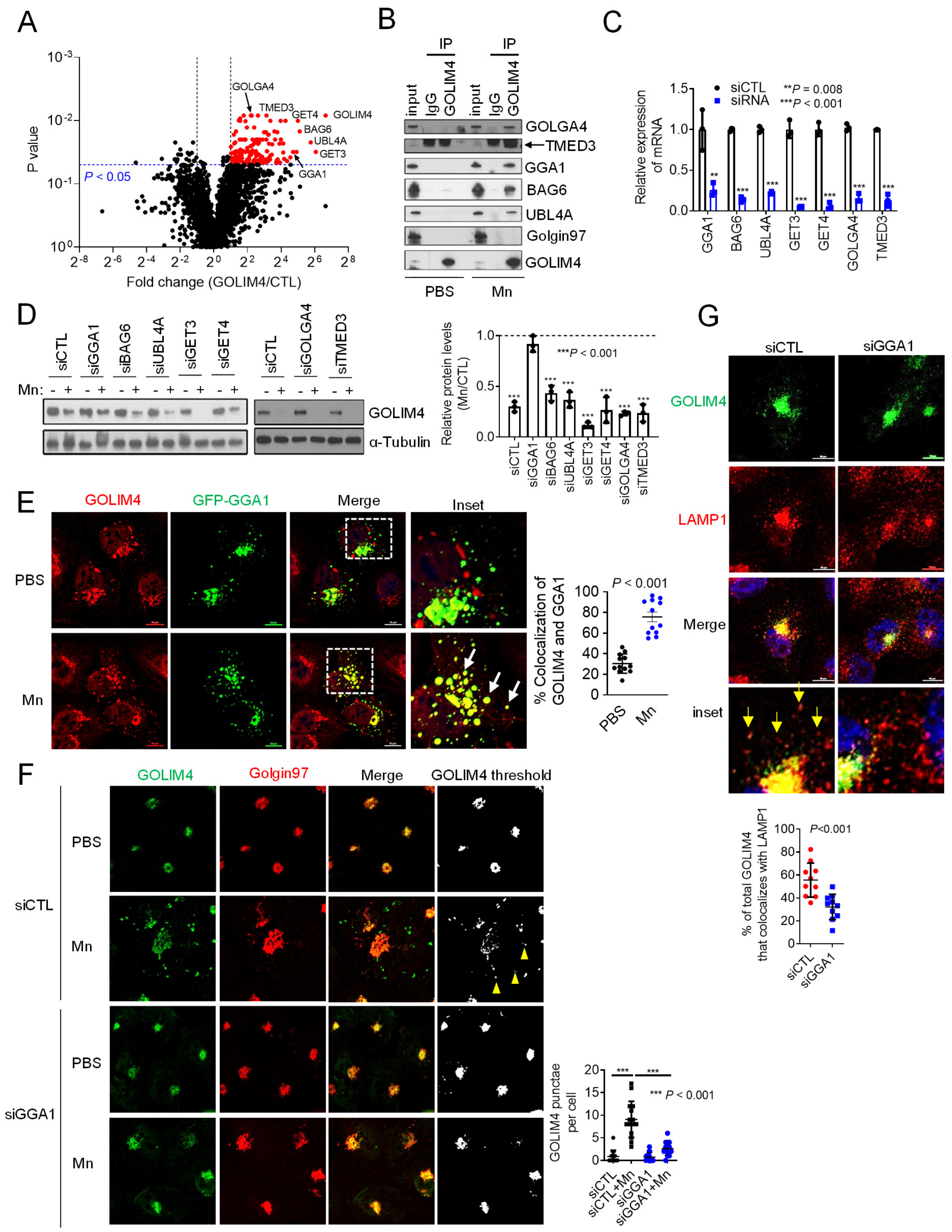

Figure S9. GGA1 mediates Mn-induced GOLIM4 translocation to lysosomes. (A) Volcano plot of proteins that associate with GOLIM4 in the presence of Mn. TURBO-ID proximity ligation assays.

Results for each protein (dot) expressed as a log2 ratio (GOLIM4/CTL, X axis) and P value (Y axis). Candidate mediators of lysosomal translocation or protein degradation are indicated. (B) IP/WB confirmation of GOLIM4-binding activity for proteins identified in (A). Golgin-97 included as a negative control. (C) qPCR confirmation of target gene depletion in siRNA-transfected H1299 cells. (D) WB analysis to determine GOLIM4 stability in the presence of Mn after depletion of GOLIM4-interacting proteins. Relative densitometric values plotted (graph). (E) Confocal micrographs demonstrate co-localization of GFP-GGA1 (green) with GOLIM4 in Mn-treated H1299 cells. Higher magnification view (inset) illustrates co-localization (arrows). Scale bar: 10 μm. Fraction of total GOLIM4 that co-localizes with GFP-GGA1 in each cell (dot, n=15). (F) Confocal micrographs demonstrate that GGA1 depletion abrogates Mn-induced translocation of GOLIM4 from Golgi. Cells were treated with or without Mn and co-stained with anti-GOLIM4 and anti-Golgin-97 antibodies. Extra-Golgi GOLIM4-staining puncta (arrow heads) were quantified per cell (n=15) and plotted (dot plot). (G) Confocal micrographs demonstrate that GGA1 depletion attenuates GOLIM4 translocation to lysosomes. Cells were treated with Mn and co-stained with anti-GOLIM4 and anti-LAMP1 antibodies. Fractions of total GOLIM4 that co-localize with LAMP1 per cell (dot, n=10) were quantified (dot plot). Data indicate the mean ± SD from a single experiment incorporating biological replicate samples (n = 3, unless otherwise indicated) and are representative of at least 2 independent experiments. P values were determined using two-tailed student's t test (for C, E, and G) or one-way ANOVA test (for D and F).

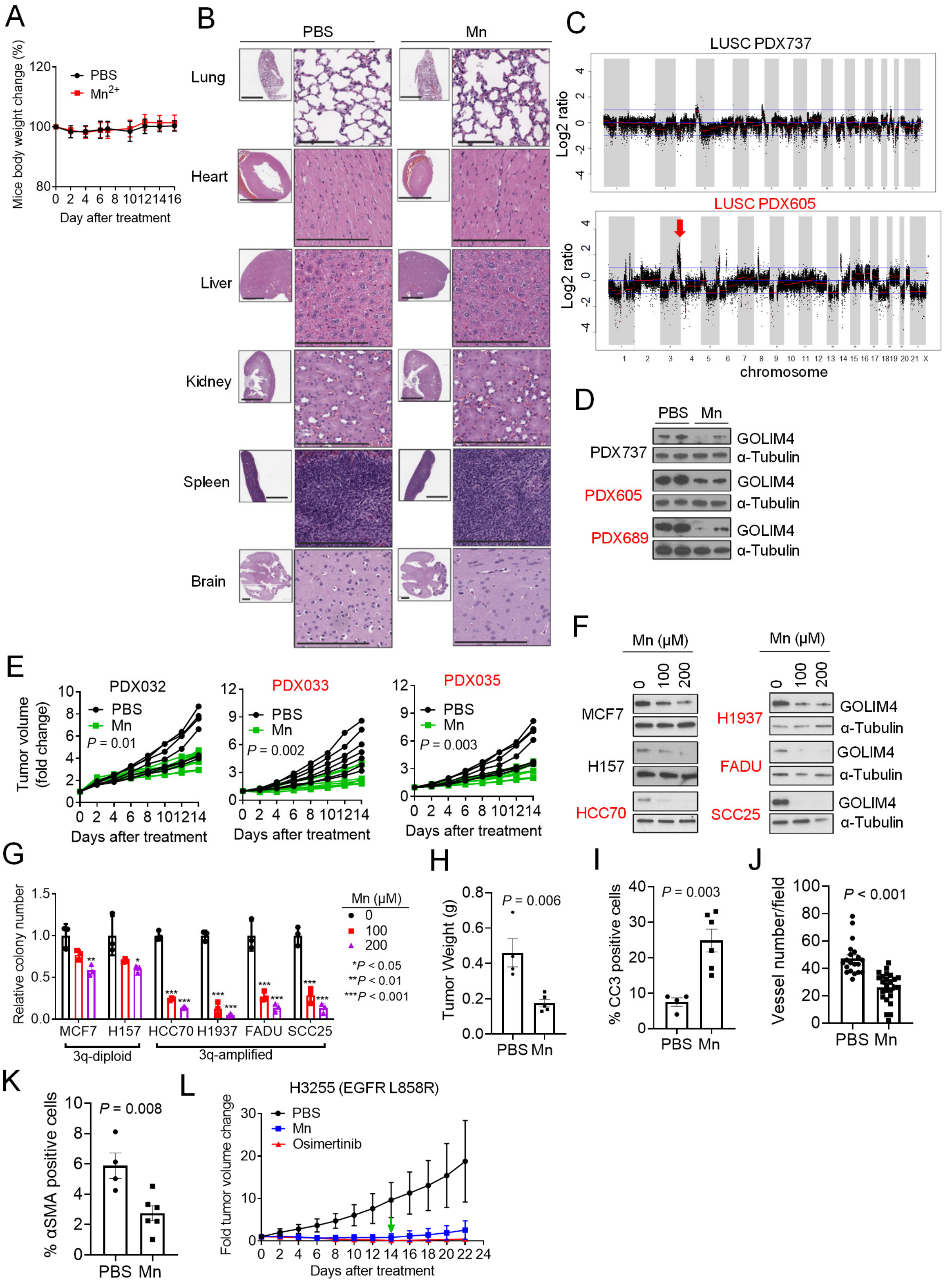
A
Mice body weight change (%)
PBS
Mn2+
Day after treatment
B
PBS
Mn
Lung
Heart
Liver
Kidney
Spleen
Brain
C
LUSC PDX737
LUSC PDX605
Log2 ratio
chromosome
D
PBS
Mn
PDX737
PDX605
PDX689
GOLIM4
α-Tubulin
E
Tumor volume (fold change)
PDX032
PDX033
PDX035
P = 0.01
P = 0.002
P = 0.003
Days after treatment
F
Mn (μM)
MCF7
H157
HCC70
H1937
FADU
SCC25
G
Relative colony number
3q-diploid
3q-amplified
*P < 0.05
**P < 0.01
***P < 0.001
H
Tumor Weight (g)
P = 0.006
I
% CC3 positive cells
P = 0.003
J
Vessel number/field
P < 0.001
K
% αSMA positive cells
P = 0.008
L
H3255 (EGFR L858R)
Fold tumor volume change
Osimertinib
Days after treatment

Figure S10. Selective vulnerability of 3q-amplified malignancies to Mn. (A) Mouse body weights during treatment normalized to day 0. (B) H&E staining of tissue sections. (C) Chromosome copy numbers in 3q-amplified (red) and -diploid (black) LUSC PDX models. 3q amplicon (arrow). (D) WB analysis of GOLIM4 levels in tumor tissues (n = 2) generated by 3q-amplified (red) or -diploid (black) LUSC PDX models. Mice treated with PBS or Mn. (E) Flank tumor weights generated by 3q-amplified (red) or -diploid (black) pancreatic ductal adenocarcinoma PDX models. Mice treated with Mn or PBS. (F) WB analysis of GOLIM4 levels in breast cancer cell lines treated with or without Mn. (G) Colony formation assay on breast and HN cancer cell lines treated with Mn. (H) Flank tumor weights. Mice injected with 344SQ cells and treated with Mn or PBS. (I-K) Intratumoral CC3+ cells (I), CD31+ vessels (J), and alpha smooth muscle actin (αSMA)+ cells (K) were quantified from IHC staining of 344SQ tumor sections. (L) Flank tumor volumes generated by EGFR-mutant H3255 LUAD cells. Mice treated with Mn or Osimertinib. Treatment stopped after 14 d (green arrow). Tumors regrew after cessation of Mn but not Osimertinib. Data indicate the mean ± SD from a single experiment incorporating biological replicate samples (n = 3, unless otherwise indicated) and are representative of at least 2 independent experiments. P values were determined two-tailed student’s t test (for A, E, H-K) or one-way ANOVA test (for G and L).

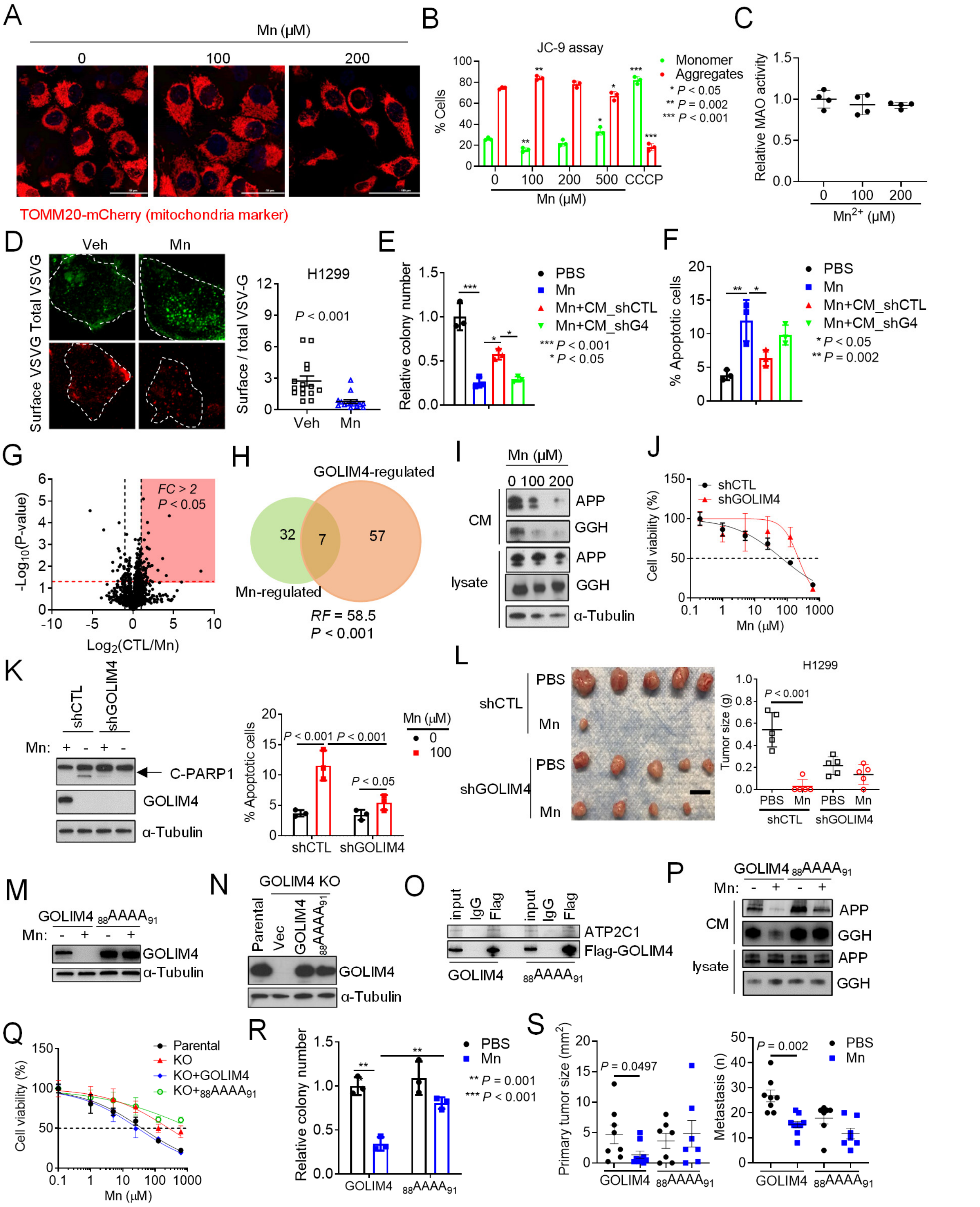

Figure S11. GOLIM4 is essential for Mn-induced anti-tumor activity. (A) Confocal micrographs detect no changes in mitochondrial morphology induced by Mn treatment. Mitochondrial marker TOMM20-Cherry. (B) Mitochondrial membrane potential assay demonstrates no evidence of membrane depolarization with Mn treatment. Carbonyl cyanide 3-chlorophenylhydrazone (CCCP)

included as a positive control for loss of membrane polarization based on disruption of membrane aggregates. (C) Monoamine oxidase (MAO) activity assay demonstrates no evidence for mitochondrial enzyme dysfunction induced by Mn treatment. (D) Single-channel and merged confocal micrographs of total and surface VSV-G in H1299 cells transfected with EGFP–VSV-G, treated with or without Mn, and imaged 30 minutes after transfer to the permissive temperature. Plot shows the ratio of surface VSV-G to total VSV-G in each cell (dot) 30 min after transfer to 32°C. Scale bar: 20 µm. (E and F) Soft agar colony formation assays (E) and Annexin V/PI flow cytometric apoptosis assays (F) on H1299 cells co-treated with Mn and CM samples from siRNA-transfected H1299 cells. (G) Volcano plot illustration of proteins identified by LC-MS analysis of CM from H1299 cells treated with or without Mn. Results expressed as a log2 ratio (Mn/resin). Y-axis: p values; X-axis: fold-change. (H) Venn diagram of secreted proteins regulated by GOLIM4 or Mn. The significance of overlap was calculated (http://nemates.org/MA/progs/overlap_stats.html). RF: representation factor. (I) WB analysis of CM samples and lysates from H1299 cells treated with or without Mn. (J) Relative cell density assays on shRNA-transfected H1299 cells treated with Mn. (K) Apoptotic cells detected by WB analysis of C-PAPR1 or Annexin V/PI flow cytometry (graph). (L) Flank tumors generated by shRNA-transfected H1299 cells. Mice treated with Mn or PBS. (M) WB analysis of *GOLIM4* KO H1299 cells reconstituted with wild-type (GOLIM4) or mutant ($_{88}$AAAA$_{91}$) GOLIM4 lacking $_{88}$DFLV$_{91}$ sequences required for Mn-binding. Cells were treated with or without Mn. (N) WB confirmation of GOLIM4 reconstitution in GOLIM4 KO H1299 cells transfected with wild-type or mutant GOLIM4. Empty vector (Vec). (O) IP/WB analysis of the cells in (M). ATP2C1-binding activity is preserved in mutant GOLIM4. (P) WB analysis of CM samples and lysates from cells in (M). Secretory activity is preserved in mutant GOLIM4-transfected cells. (Q and R) Relative cell density assay (Q) and soft agar colony formation assay (R) on cells generated in (M) and treated with Mn. (S) Primary tumor size (left) and metastasis numbers (right) generated by intrathoracically injected H1299 transfectants. Mice were treated with or without Mn. Data indicate the mean ± SD from a single experiment incorporating biological replicate samples (n = 3, unless otherwise indicated) and are representative of at least 2 independent experiments. P values were determined using two-tailed student's t test (for D, J, K, L, and S) or one-way ANOVA test (for B, C, E, F, Q, and R).

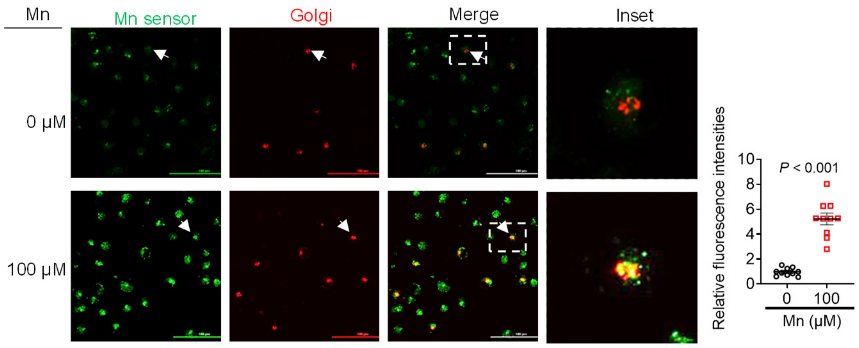

Figure S12. Validation of Mn sensor. Single-channel and merged confocal micrographs of Mn sensor M1 (green) in H1299 cells. CellLight Golgi-RFP (red). Higher magnification view of merged images (inset). Scale bar: 100 µm. Relative fluorescent intensity of Mn sensor M1 per cell was quantified (bar graph). Data indicate the mean ± SD from a single experiment incorporating biological replicate samples (n = 10). P values were determined using two-tailed student's t test.
