# Supplementary material for: Chromosomal 3q amplicon encodes essential regulators of secretory vesicles that drive secretory addiction in cancer
Source: J Clin Invest. 2024 Apr 25;134(12):e176355. doi: 10.1172/JCI176355 (PMC11178546; doi:10.1172/JCI176355)

Fig. 2C

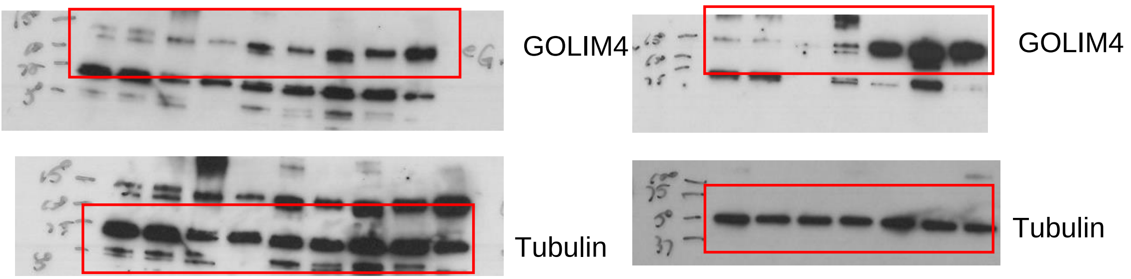

Fig. 2D

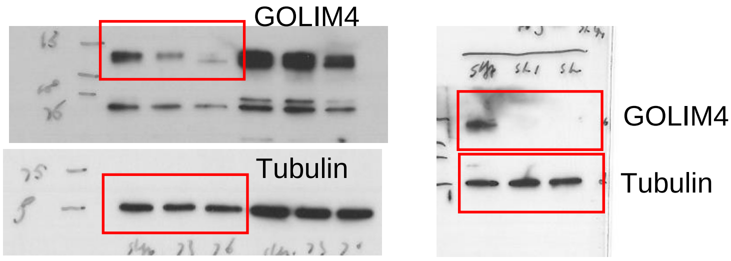

Fig. 2H

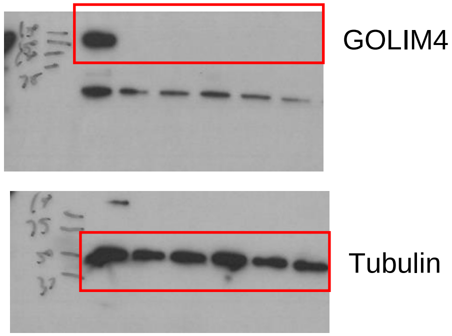

Fig. 3G

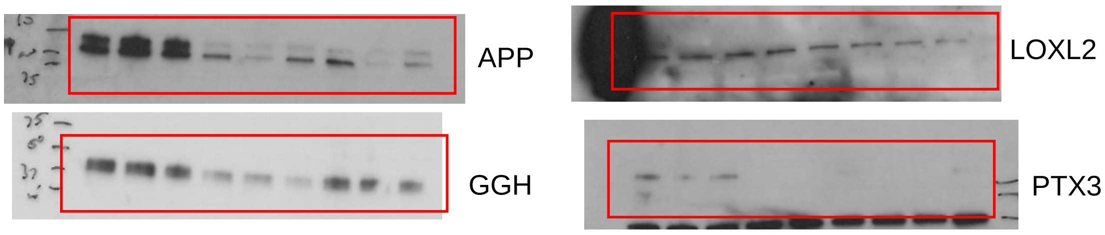

Fig. 3H

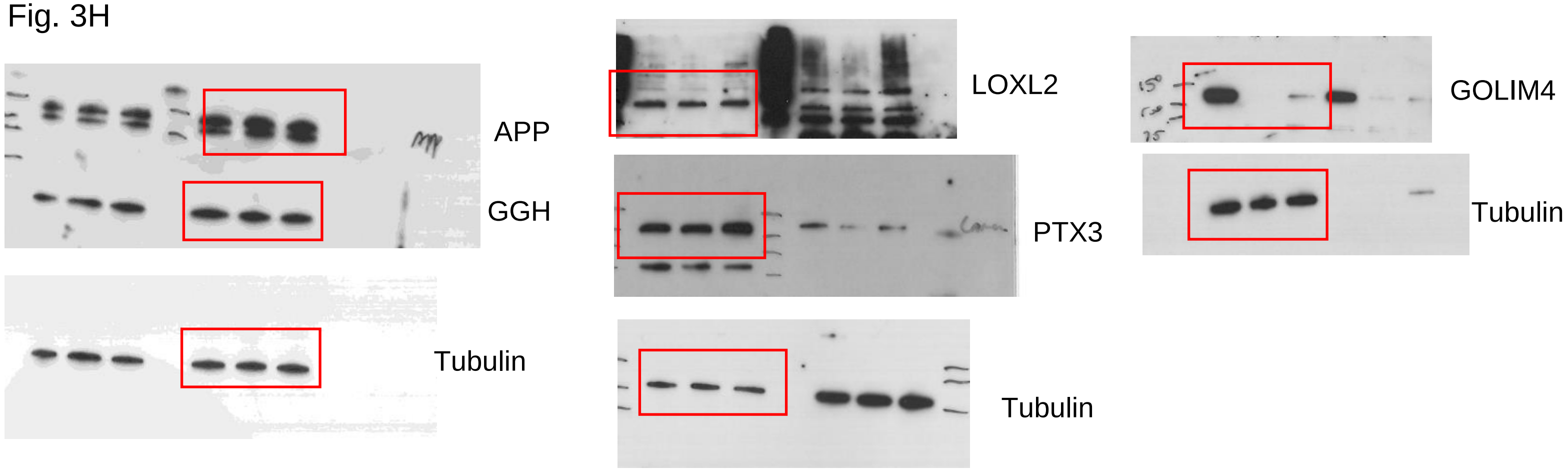

Fig. 4C

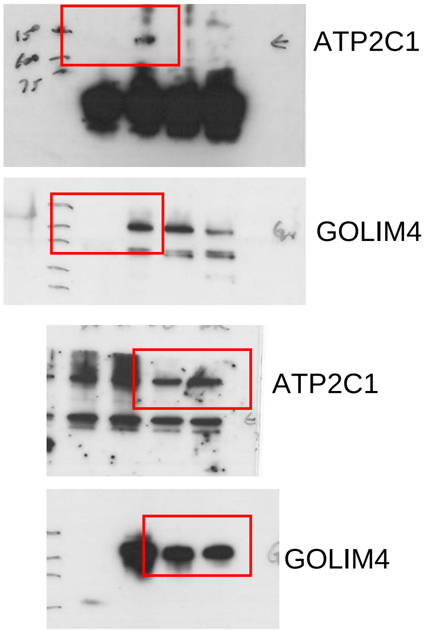

Fig. 4H

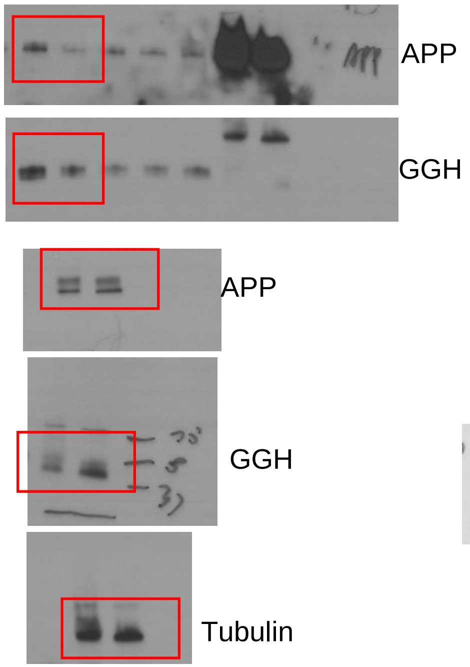

Fig. 4I

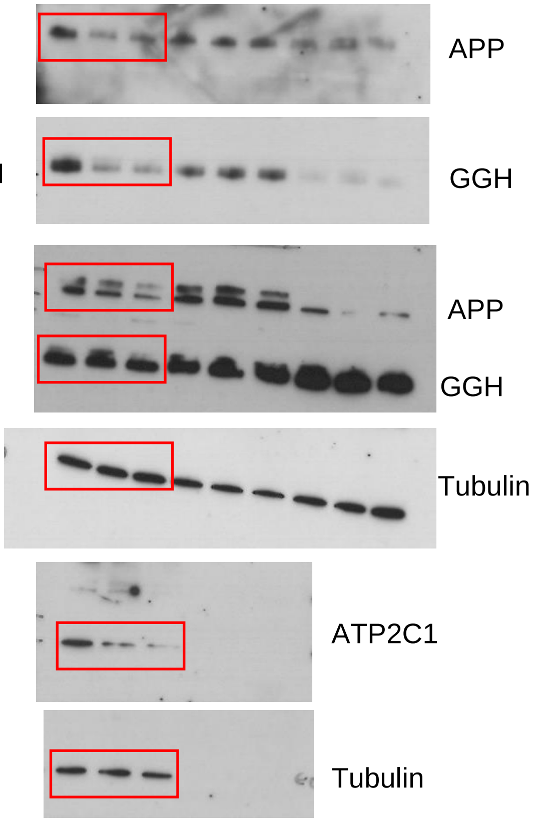

Fig. 4J

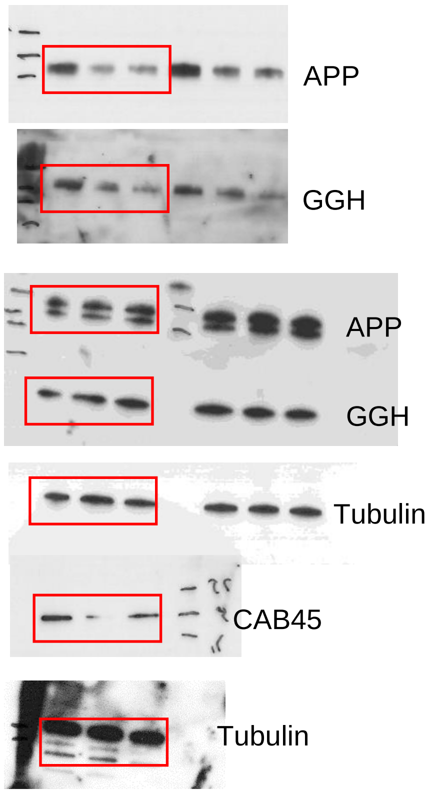

Fig. 4Q

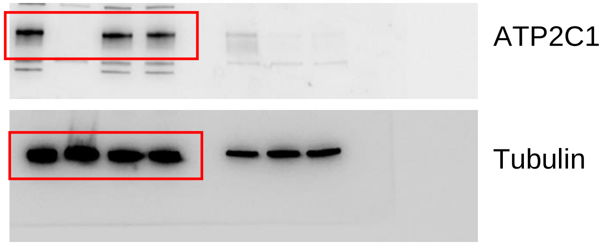

Fig. 5B

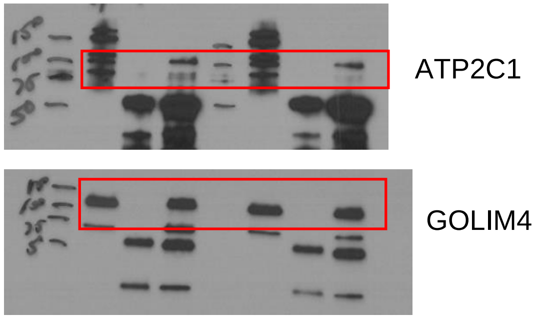

Fig. 5J

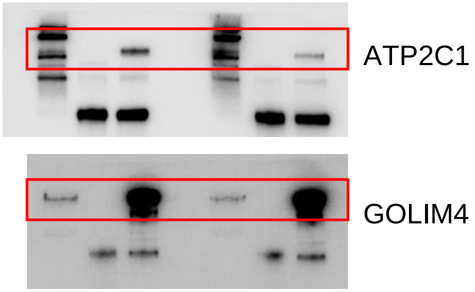

Fig. 5C

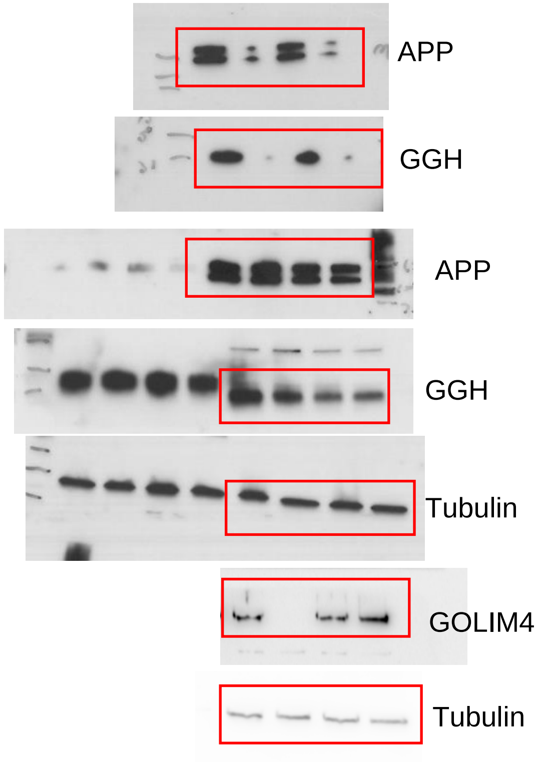

Fig. 5E

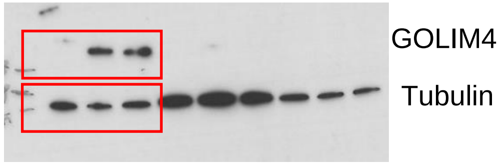

Fig. 5I

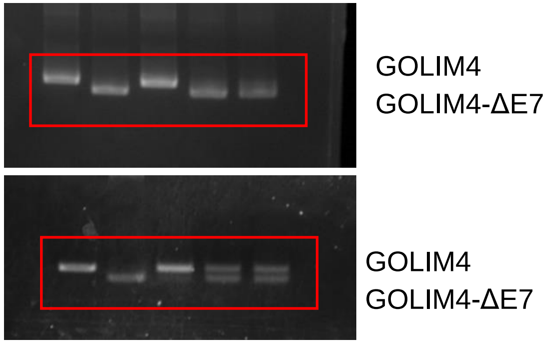

Fig. 6A

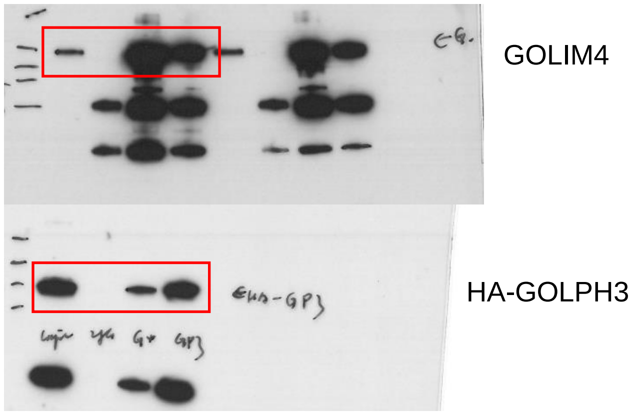

Fig. 6C

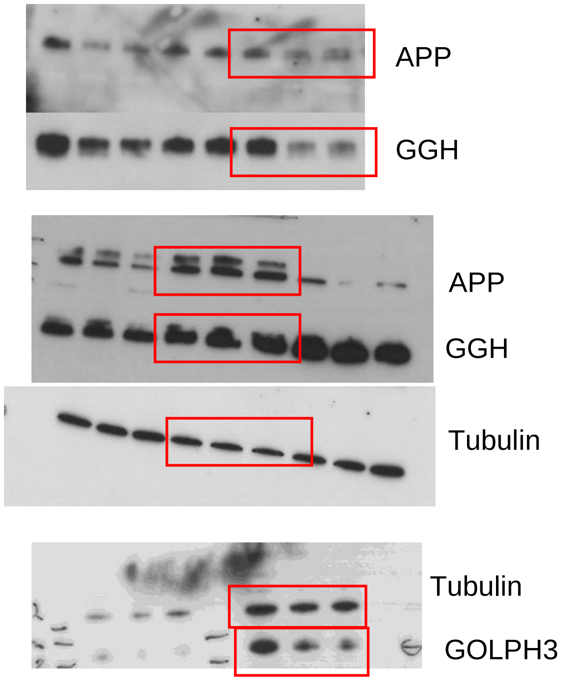

Fig. 6F

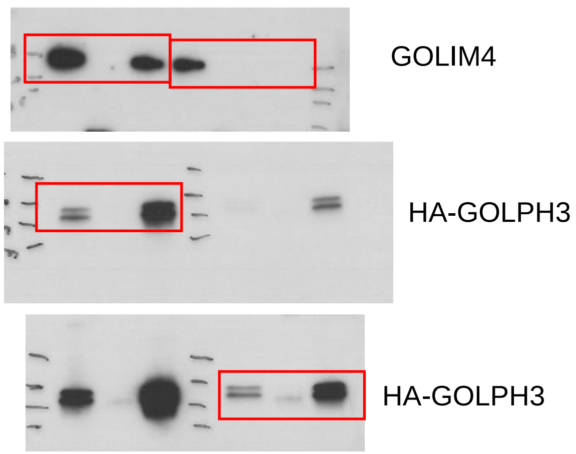

Fig. 6G

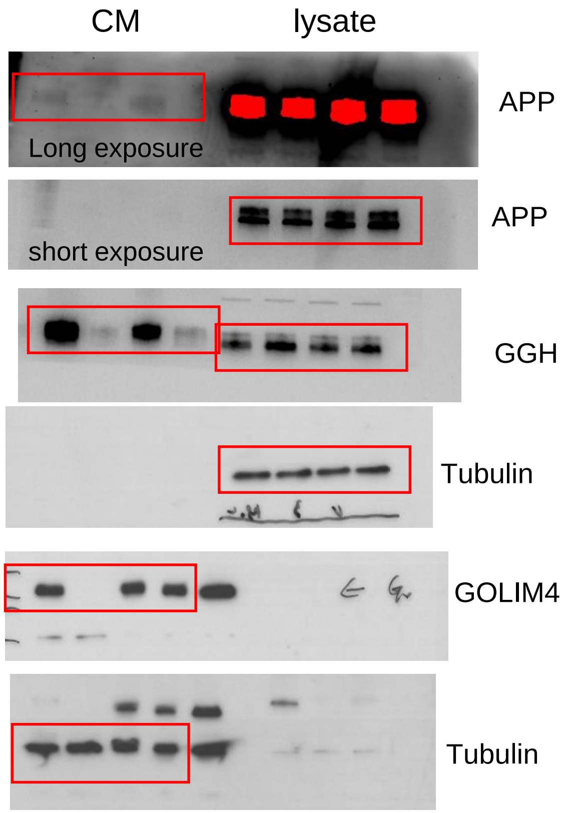

Fig. 7A

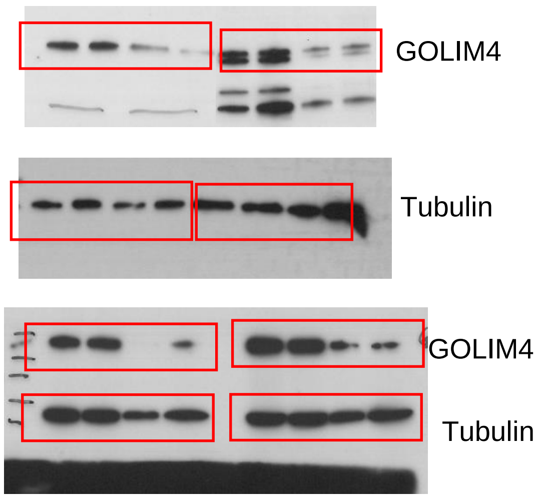
GOLIM4
Tubulin
GOLIM4
Tubulin

Fig. 7F

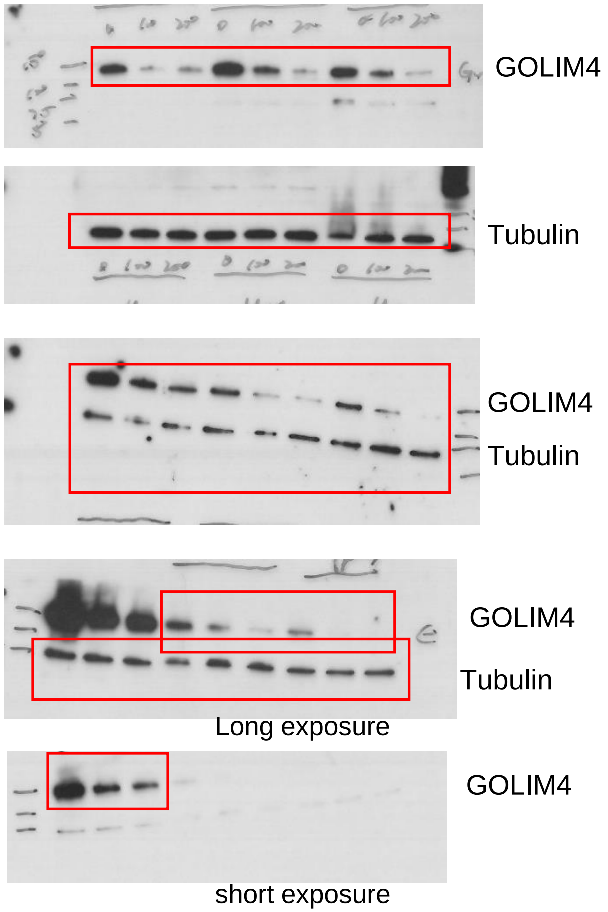
GOLIM4
Tubulin
GOLIM4
Tubulin
GOLIM4
Tubulin
Long exposure
GOLIM4
short exposure

Fig. 8A

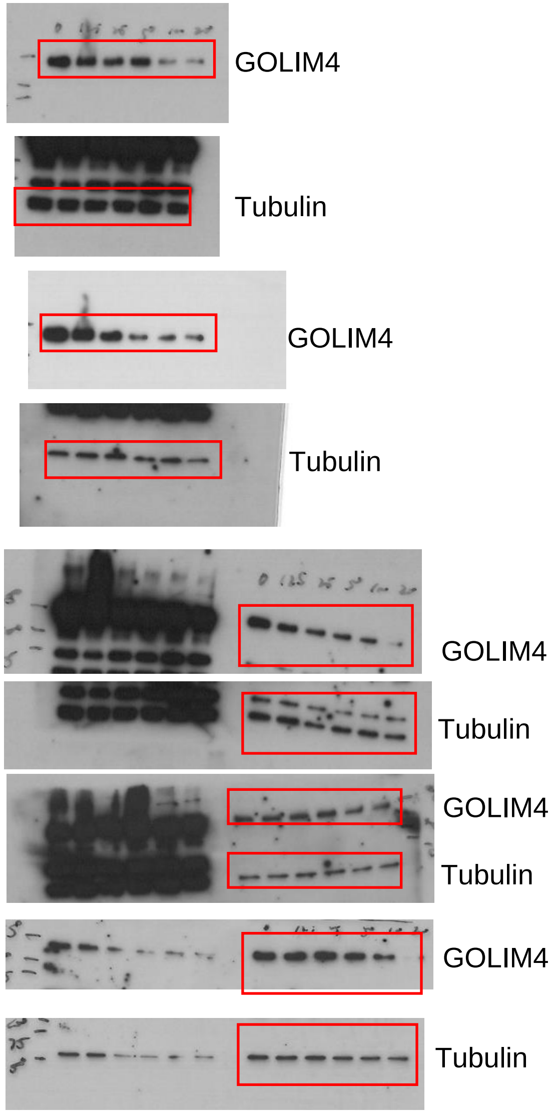

Fig. 8B

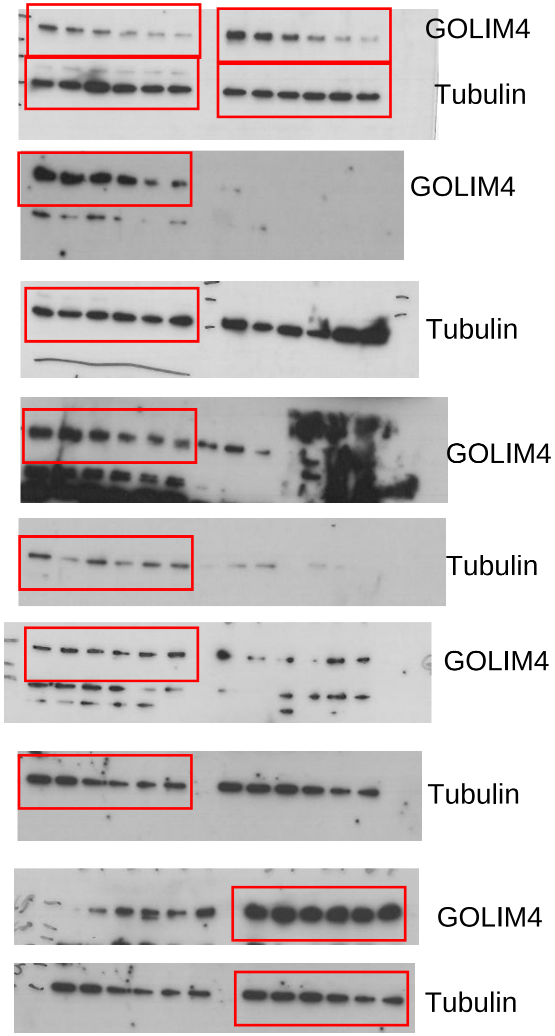

Fig. 8F

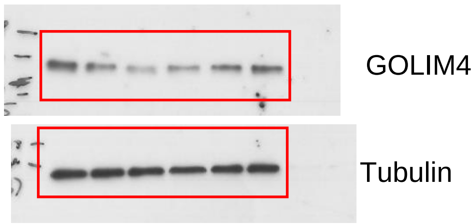

Fig. 8G

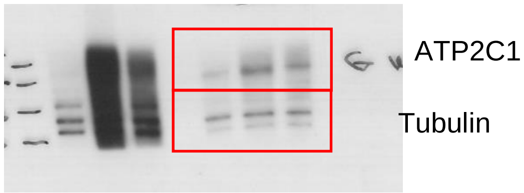

Fig. 8I

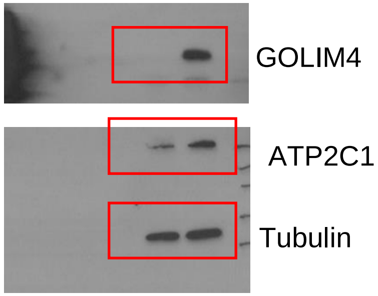

Fig. S1E

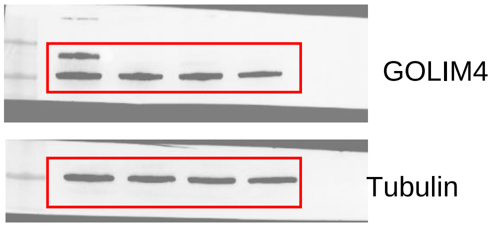

Fig. S1H

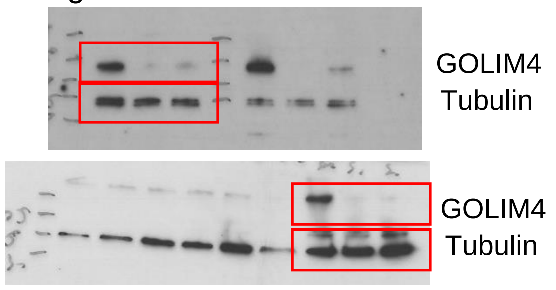

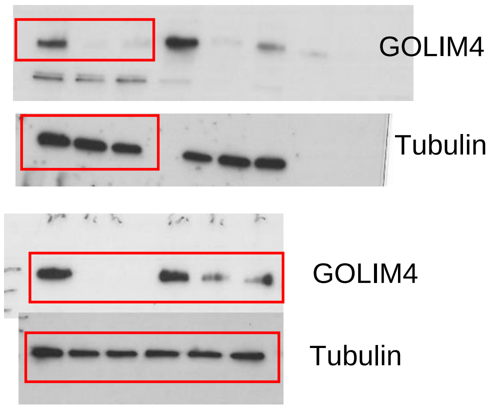

Fig. S1J

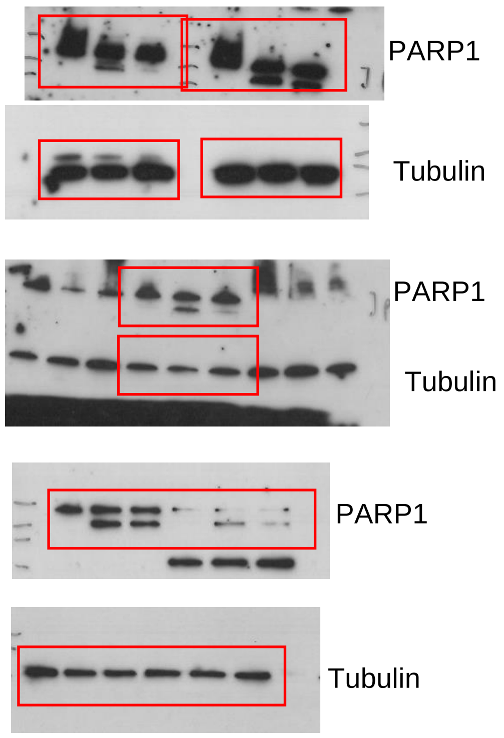

Fig. S1P

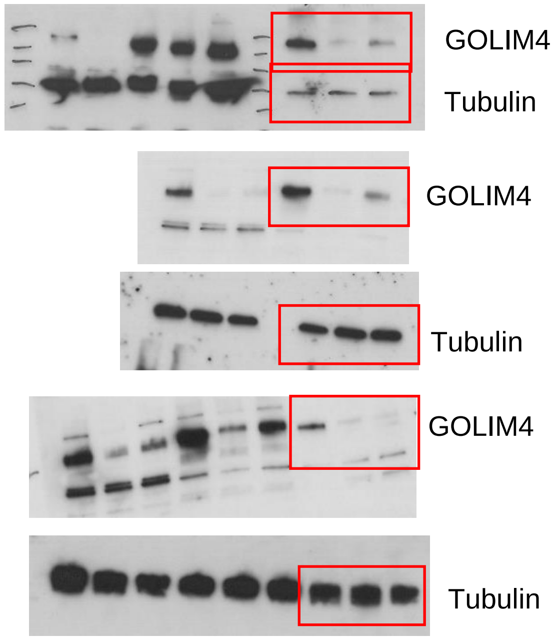

Fig. S2C

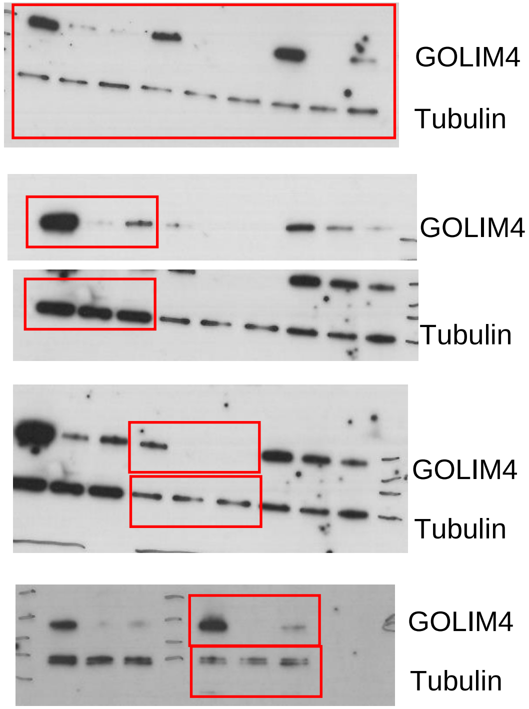

Fig. S2G

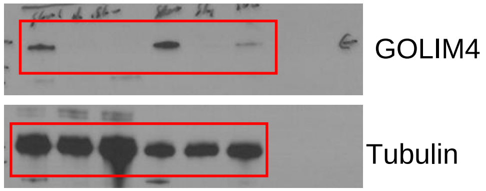

Fig. S3E

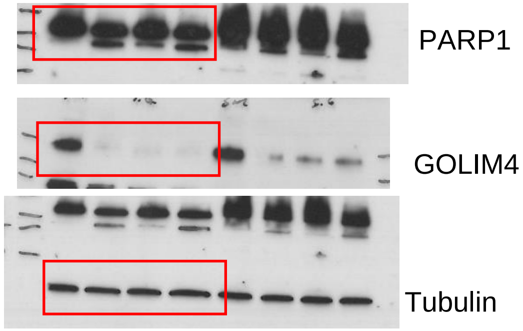

Fig. S3F

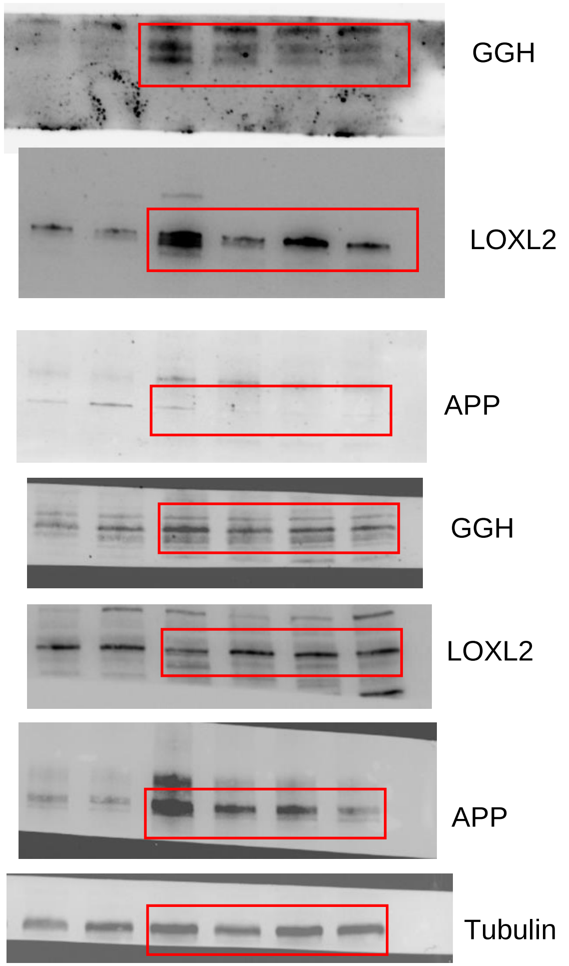

Fig. S3G

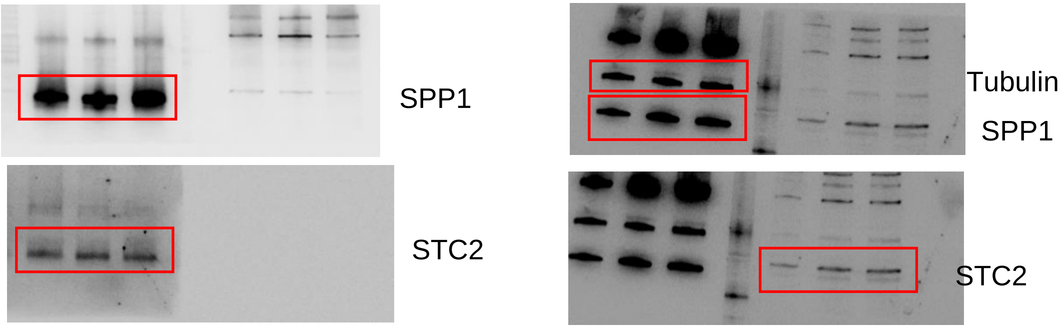

Fig. S4D

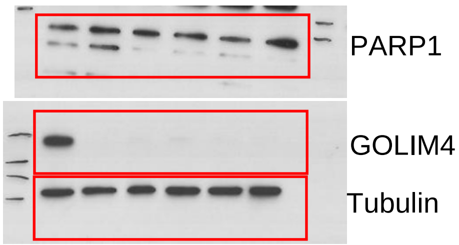

Fig. S4F

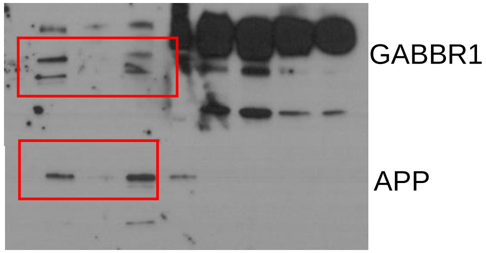

Fig. S4H

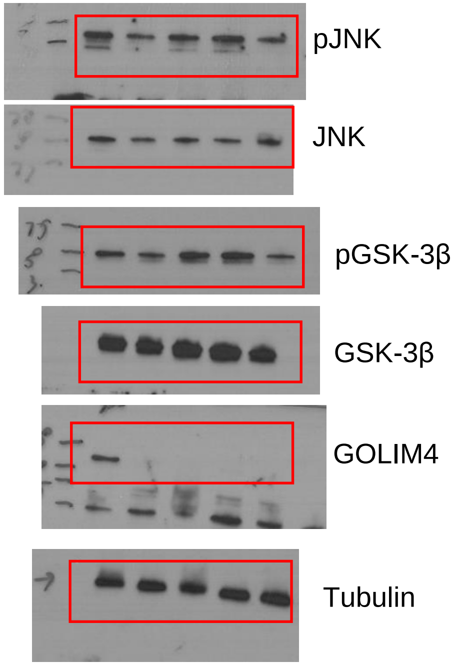

Fig. S6A

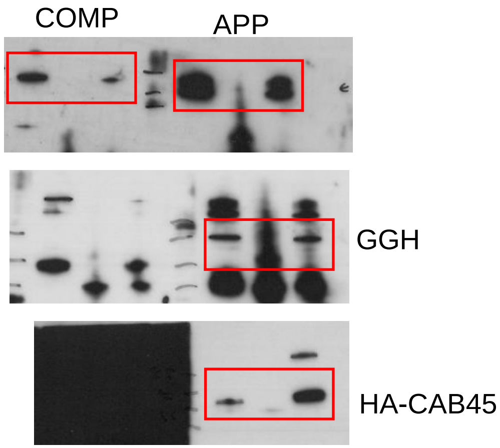

Fig. S6I

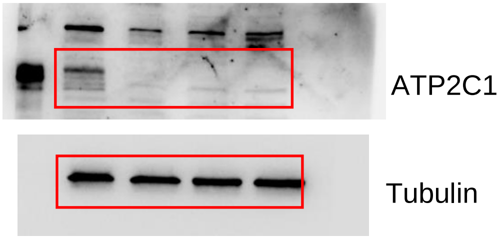

Fig. S7A

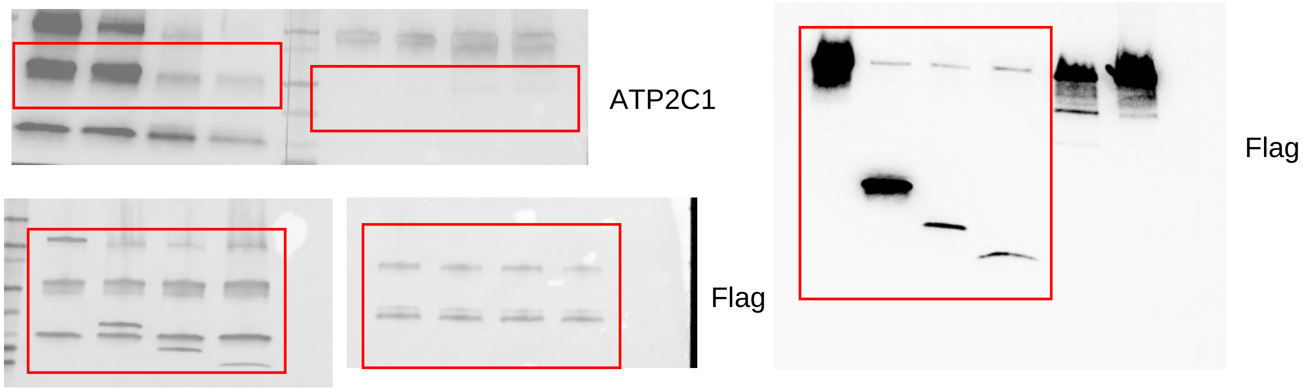

Fig. S8A

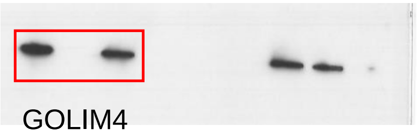

Fig. S8D

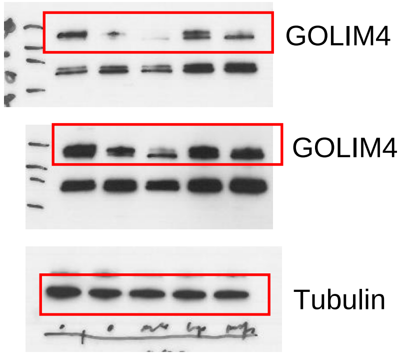

Fig. S9B

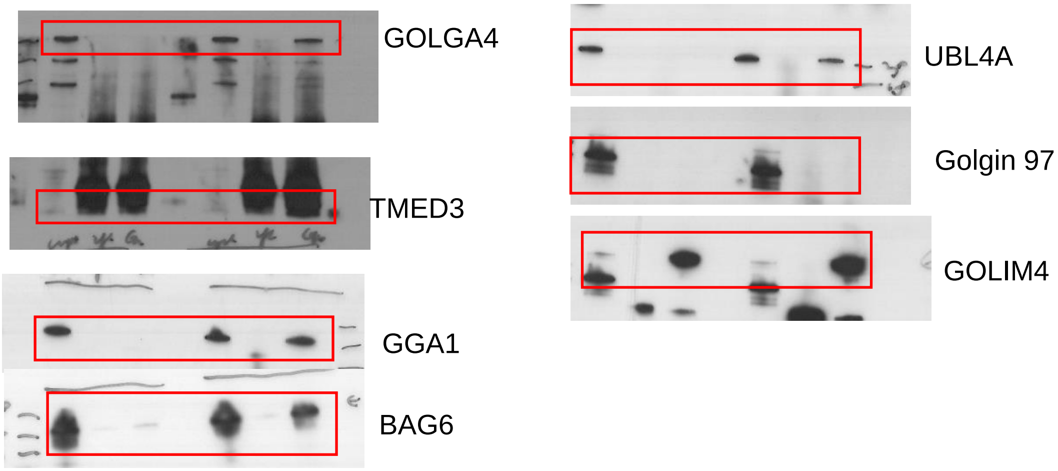

Fig. S9D

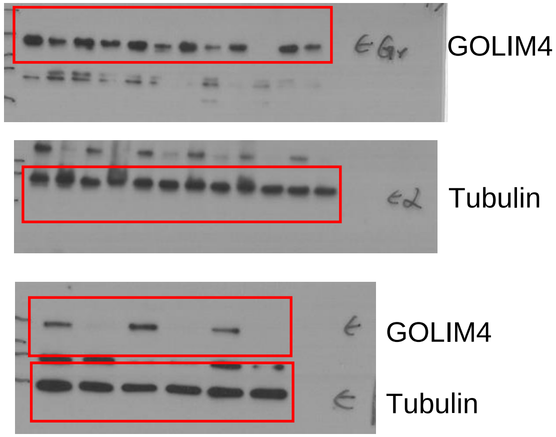

Fig. S10D

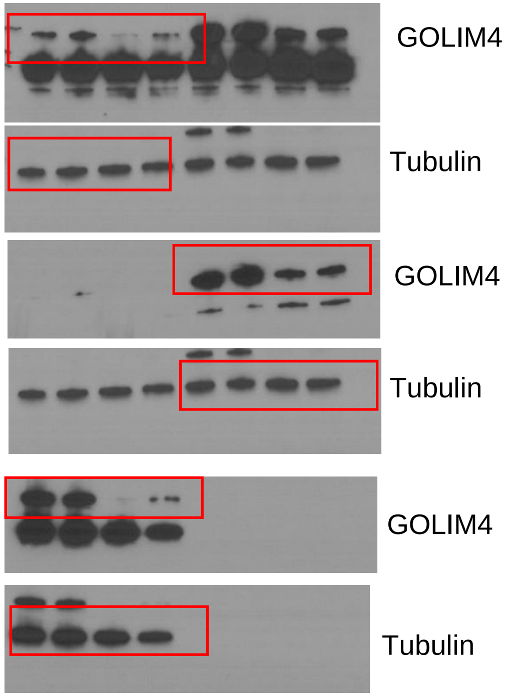

Fig. S10F

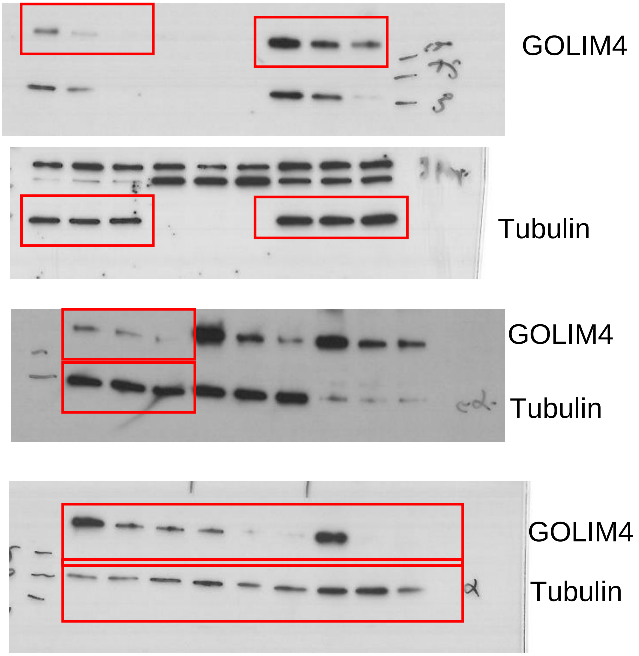

Fig. S11I

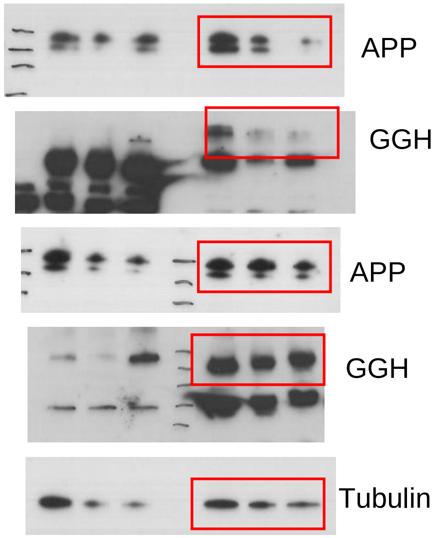

Fig. S11K

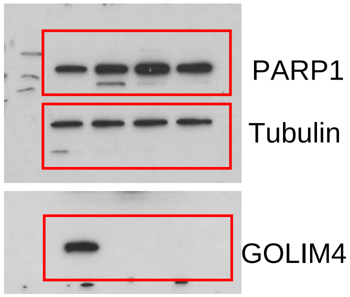

Fig. S11M

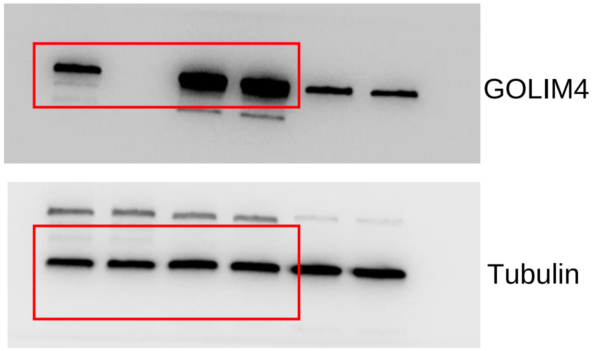

Fig. S11N

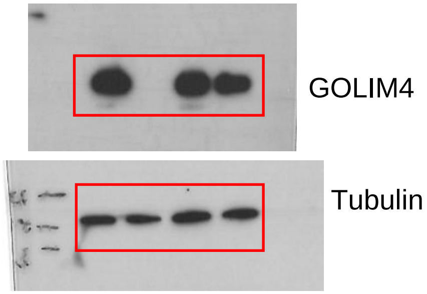

Fig. S11O

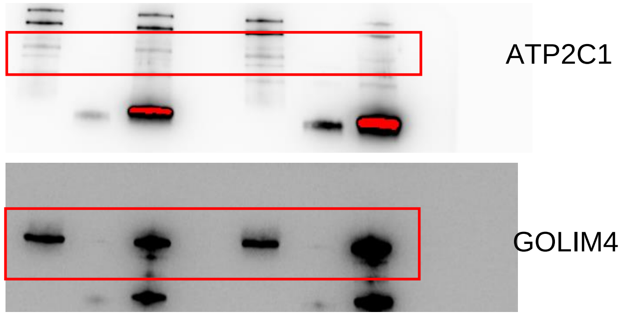

Fig. S11P

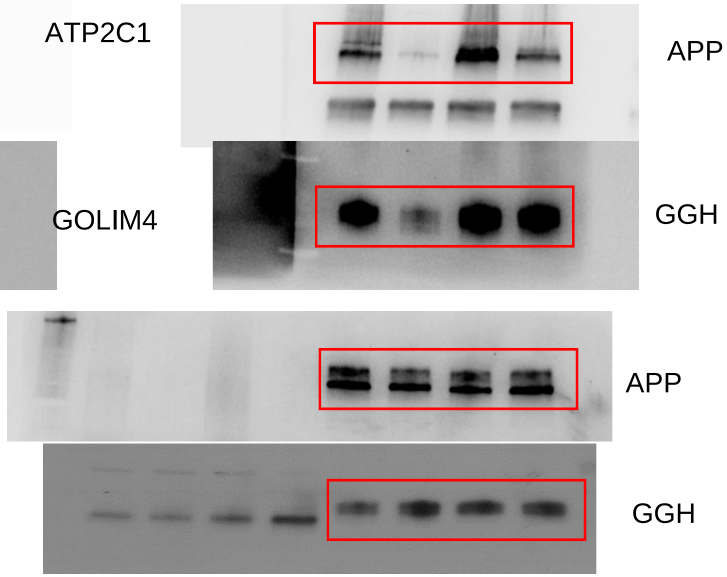

Supplement: Unedited blot and gel images [file jci-134-176355-s235.pdf]
