# Supplementary material for: Chromosomal 3q amplicon encodes essential regulators of secretory vesicles that drive secretory addiction in cancer
Source: J Clin Invest. 2024 Apr 25;134(12):e176355. doi: 10.1172/JCI176355 (PMC11178546; doi:10.1172/JCI176355)
Supplement: Supplemental table 4 [file jci-134-176355-s239.pdf]

Table S4. Primer sequences.

qPCR primers		
**Gene**	**Forward (5′-3′)**	**Reverse (5′-3′)**
BAG6	TGCTGCCTTCATACAACGCCTC	GTCCACCATAGAGAAGTTCTGGC
GGA1	TCACGGAGATGGTGATGAGCCA	TCCTCTGTGTCACTCGCCAGTC
UBL4A	TGGTCTCCGAGAAGCTGAACGT	GACCACTAGGTTGAGCTTGGAG
GET3	GTCAGCGAACAGTTCAAGGACC	TCCTGGATCAGCCTCTCTGTCT
GET4	CGTTTGTGGAGCCGCTGCTTAA	GATGGCTGGTACTGCTCACACA
TMED3	TCCACCTTCTCTCACAAGACCG	CCGTTTTCAGAGCCTCATGGATG
GOLGA4	AGTCGGGTGAAACAGGAGGTTG	GCTTCTTGGTCAGTTCCTGCTC
GABBR1	CCTGAACAAGACATCTGGAGGAG	GCTGGCATCAAACACCACATGG
FBLN1	GTGGCTACCATCTCAACGAGGA	CTTGCATTCGCAGCGGAAACTG
SERPINE2	ATCCAGCCTCTGCCTGTGATTC	TTGACGAGGACCAGTCTGGTGA
CLEC11A	ACACCCGCGATGCCGTGCAAG	CGAGAGCAGGAAGCACTTGTGG
GGH	TGCAGGTGCGAGAGTTGTACCA	GAGCGTCTGAGGTCAACACTTC
HTRA1	CAGACGTGATCTCAGGAGCGTA	TCGCTGACATCATTGGCGGAGA
LAMC2	TACAGAGCTGGAAGGCAGGATG	GTTCTCTTGGCTCCTCACCTTG
LOXL2	TGACTGCAAGCACACGGAGGAT	TCCGAATGTCCTCCACCTGGAT
APP	CCTTCTCGTTCCTGACAAGTGC	GGCAGCAACATGCCGTAGTCAT
GOLIM4	ATGAGCCTCGTGAACAAGGACC	CTCTCACTTGCTCGGCTTCTTC
ATP2C1	GGAACACTGGTCAGATGTGGCA	AGAGGTCCATGCTCTTCTGCAG
RBFOX2	CCAGCTTTCAAGCAGATGTGTCC	CAAATGGGCTCCTCTGAAAGCG
GOLIM4 (gDNA)	ATGAGCCTCGTGAACAAGGACC	CATCACATACACGTACACCATGG
**RT-PCR primers**		
GOLIM4 (Exons 6-8)	GCACACCAAGACATACATACACAG	CTTCTCCCTTGCTGTGTTGTAAC
**Clone primers**		
**Primers for gene expression (pcDNA3.1)**		
GOLIM4 (HA tag)	CCGCTCGAGATGTACCCATACGATGTTCCAGATTACGCTATGGGAAACGGGATGTGCTCCCGAAAGCAGAAGCGGGGATCCCCT	GGGGATCCCTACATTTCAGCTCTTCGATGTGA
GOLIM4 (KR mut)	CCGCTCGAGATGGGAAACGGGATGTGCTCCCGAAAGCAGgcGgcGATTTTCCAGACGCTGCTGC	GGGGATCCCTACATTTCAGCTCTTCGATGTGA
GOLIM4 (DFLV mut)	ATTTAATGTTTCTTGTGCTTCTAACTTATAAGCAGCAGCAGCTTCCTTTGCTTTTTTATGTTCAAG	CTTGAACATAAAAAAGCAAAGGAAGCTGCTGCTGCTTATAAGTTAGAAGCACAAGAAACATTAAAT
GOLIM4 (Flag tag)	CCGCTCGAGATGGACTACAAAGACGATGACGACAAGATGGGAAACGGGATGTGC	GGGGATCCCTACATTTCAGCTCTTCGATGTGA
GOLIM4 (1-245)	CCGCTCGAGATGGACTACAAAGACGATGACGACAAGATGGGAAACGGGATGTGC	GGGGATCCTTATGGAATCCTATTCAGAGTATCTTTCAG
GOLIM4 (1-175)	CCGCTCGAGATGGACTACAAAGACGATGACGACAAGATGGGAAACGGGATGTGC	GGGGATCCTTAATACAGTCTCTTTTAGCTTAGAAAGTGTTCTT
GOLIM4 (1-87)	CCGCTCGAGATGGACTACAAAGACGATGACGACAAGATGGGAAACGGGATGTGC	GGGGATCCTTATTCCTTTGCTTTTTTATGTTCAAGT
CAB45 (HA tag)	GCAGAATTCATGGTCTGGCCCTGGGT	CGTGGATCCAGCGTAGTCTGGGACGTCGTATGGGTAAAACTCCTCGTGCACGCTG
**Primers for gene expression (pRFP-C3)**		
GOLIM4	CCGCTCGAGATGTACCCATACGATGTTCCAGATTACGCTATGGGAAACGGGATGTGCTCCCGAAAGCAGAAGCGGGGATCCCCT	GGGGATCCCTACATTTCAGCTCTTCGATGTGA
**Primers for gene expression (TurboID)**		
GOLIM4 (TurboID)	CCGCTCGAGATGTACCCATACGATGTTCCAGATTACGCTATGGGAAACGGGATGTGCTCCCGAAAGCAGAAGCGGGGATCCCCT	GCTCTAGACTACATTTCAGCTCTTCGATGTGA
